# miR-30c plays diagnostic and prognostic roles and mediates epithelial–mesenchymal transition (EMT) and proliferation of gliomas by affecting Notch1

**DOI:** 10.1038/s41598-022-19326-x

**Published:** 2022-09-30

**Authors:** Mengkao Li, Wenzhi Liu, Jian Li, Hong Zhang, Jin Xu

**Affiliations:** 1 Department of Neurosurgery, People’s Hospital of Longhua, Shenzhen, Guangdong Province People’s Republic of China; 2 Department of Radiation Oncology, Shenzhen Traditional Chinese Medicine Hospital, Shenzhen, Guangdong Province People’s Republic of China; 3 The Fourth Clinical Medical College of Guangzhou University of Chinese Medicine, Shenzhen, Guangdong Province People’s Republic of China; 4 Department of Neurosurgery, Taian Central Hospital, Taian, Shandong Province People’s Republic of China; 5 Department of Clinical Oncology, Taian Central Hospital, Taian, Shandong Province People’s Republic of China; 6 Department of Radiation Oncology, Shandong Cancer Hospital and Institute, Shandong First Medical University and Shandong Academy of Medical Sciences, Jinan, Shandong Province People’s Republic of China

**Keywords:** Cancer, Molecular biology

## Abstract

miR-30c functions as a tumor suppressor gene in the majority of tumors, including gliomas. In our study, we discovered that the expression levels of miR-30c in glioma tissues and plasma prior to surgery were lower than those in normal brain tissue following brain injury decompression and in plasma in healthy volunteers. The low expression of miR-30c was closely aligned with the WHO grade, tumor size, PFS, and OS. Additionally, the miR-30c expression level in tumor tissue was positively correlated with the levels in preoperative plasma. In cell biology experiments, miR-30c inhibited EMT and proliferation, migration, and invasion of glioma cells. Analysis of databases of miRNA target genes, real-time quantitative PCR, western blotting, and dual luciferase reporter assays demonstrated that Notch1 is the direct target gene of miR-30c. An inhibitor and shRNA-Notch1 were cotransfected into glioma cells, and it was found that shRNA-Notch1 reduced the enhancement of inhibitors of EMT and proliferation, migration, and invasion of glioma cells. Therefore, we believe that when utilized as a tumor suppressor gene, miR-30c can inhibit EMT and the proliferation, migration, and invasion of glioma cells by directly acting on Notch1 at the posttranscriptional level and that it is a potential diagnostic and prognostic marker.

Gliomas are among the most common malignant tumors of the central nervous system that arise from glial cells or precursor cells. According to World Health Organization (WHO) classifications, gliomas are categorized into four grades. Grade 1 and grade 2 gliomas are considered low grade, and grade 3 and grade 4 gliomas are high grade. Grade 4 gliomas are labeled glioblastomas (GBMs). Despite active and effective treatment, the median survival time of GBM patients is less than 20 months^1^. Therefore, the treatment of gliomas poses a great challenge. To establish effective treatment methods, clarifying the molecular mechanisms associated with the occurrence and development of gliomas is paramount.

Epithelial mesenchymal transition (EMT) is defined as the biological process through which epithelial cells acquire a mesenchymal phenotype^2^. In the EMT process, epithelial cells lose cell polarity, decrease contact with surrounding cells and stromal cells, decrease interaction between cells, enhance cell migration and mobility, alter the cell phenotype, and lose the epithelial phenotype. Regarding the latter, one such example is a decrease in E-cadherin levels, which leads to decreased cell adhesion^3^ and causes cells to obtain the characteristics of invasion and metastasis^4^. Simultaneously, the number of cells that obtain mesenchymal phenotypes, including the presence of Vimentin and N-cadherin, is increased^5,6^.

The Notch signaling pathway is a common signaling pathway involved in EMT process control^7,8^. The Notch signaling pathway comprises the Notch receptor, Notch ligand, CSL-DNA binding protein, and Notch regulatory molecules. A signal transmitted between adjacent cells through the Notch receptor can regulate cell differentiation, proliferation, and apoptosis. The Notch receptor is a single transmembrane protein encoded by the Notch gene. Four kinds of Notch receptors (Notch 1, 2, 3, and 4) are located in mammals, including humans. Notch signaling is activated by the binding of Notch ligands to the corresponding receptors of adjacent cells. The receptors are subsequently hydrolyzed by tumor necrosis factor alpha converting enzyme (TACE) of the metalloproteinase family and release the N-terminal and C-terminal fragments. Then, the N-terminal fragment (extracellular region) is internalized into the ligand-expressing cells, and the C-terminal fragment (intracellular region) is secondarily hydrolyzed by γ-secretase, releasing the Notch intracellular domain (NICD). NICD finally enters the nucleus and binds with recombinant signal binding protein J κ (RBP-J κ) to activate the transcription of downstream target genes^9^. Therefore, the formation of NICD is a marker of Notch signaling pathway activation^10^. The target genes downstream of the Notch signaling pathway belong to the HES and HEY families, in which HES1 and HEY1 are mainly expressed on endothelial cells, and their expression level is often used as an important index for the activation of the Notch signaling pathway^11^.

In the process of malignant tumorigenesis, the Notch signaling pathway plays carcinogenic roles, leading to cell proliferation^12^, differentiation^13^, apoptosis^14^, and process imbalance, all of which contribute to the occurrence and development of malignant tumors. In gliomas, Notch1 is highly expressed^15^. In glioma cells, Notch1 expression is upregulated and plays a role in tumor progression. Notch1 may also function as an independent prognostic marker that is both separate from and supplementary to the WHO classifications^16^. Furthermore, Notch1 promotes EMT in a variety of tumors, including gliomas^17–19^.

miRNAs are 19–22-nucleotide-long noncoding RNAs that act as oncogenes or tumor suppressor genes by connecting to the 3′-UTR of a target gene and regulating the expression of related proteins at the posttranscriptional level. miRNAs function in regulating cell proliferation^20^, apoptosis^21^, invasion^22^, angiogenesis^23^, and the cell cycle^24^ and in turn participate in the occurrence and progression of an assortment of tumors. miR-30c plays different roles in different tumors^25–27^, but there is no comparable research into the role miR-30c in gliomas. In this study, the mechanism of miR-30c in gliomas is explored, and a theoretical basis for the diagnosis and treatment of gliomas is presented.

## Results

### The expression of miR-30c was decreased in tissues and plasma from patients with gliomas

**Figure 1:**
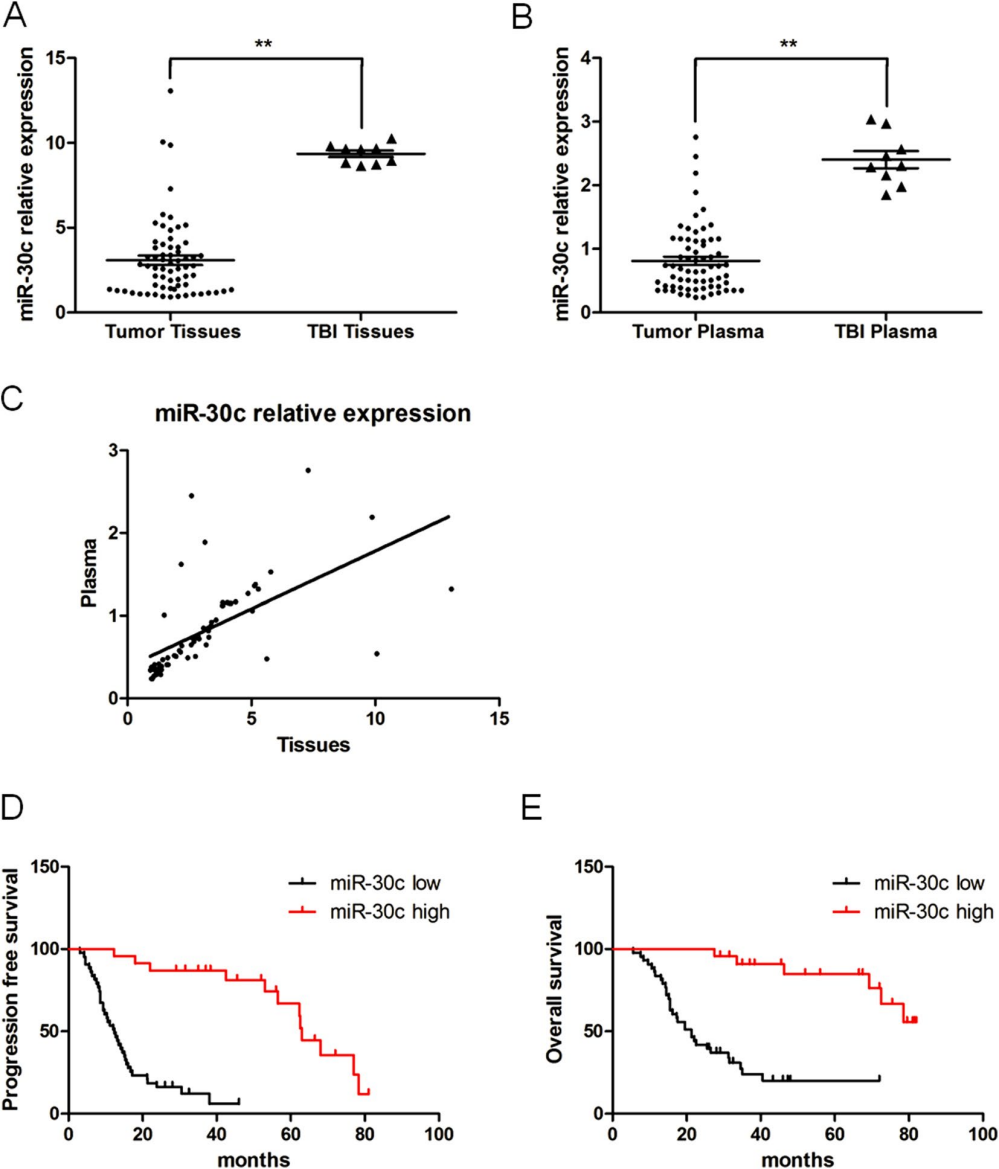
The expression levels of miR-30c in human glioma tissue, preoperative plasma, and paraffin specimens. (**A**) The expression levels of miR-30c in glioma tissues were significantly lower than the levels in normal brain injury tissue (**P < 0.01). (**B**) The expression levels of miR-30c in the plasma of patients with glioma prior to operation were significantly lower than the levels in healthy volunteers (**P < 0.01). (**C**) Correlation analysis of miR-30c in glioma tissue and plasma. (**D**, **E**) The PFS and OS of patients with high miR-30c expression were significantly longer than those of patients with low miR-30c expression (*P* < 0.01).

The expression of miR-30c was determined using qRT‒PCR. As detailed in Fig. 1, the relative expression level of miR-30c in 66 glioma tissues was 3.077 ± 0.282, while the relative expression level of miR-30c in 9 patients with traumatic brain injury was 9.352 ± 0.190. The expression levels of miR-30c in glioma tissues were significantly lower than those in normal brain injury tissues (*P* < 0.01) (Fig. 1A). As detailed in Table 1, the expression level of miR-30c in glioma tissues was closely correlated with the WHO grade and tumor size (*P* < 0.01) but not with the sex, age, extent of resection, or KPS scores of the patients (*P* > 0.05).

**Table 1:** The connection between miR-30c levels in tissue and plasma and the clinicopathologic characteristics of glioma patients (* Indicates *P* < 0.05, ** Indicates *P* < 0.01).

Characteristics	Number	Tissue	*P* value	Plasma	*P* value
**Gender**					
Male	41	3.132 ± 0.287	0.806	0.804 ± 0.068	0.865
Female	25	2.987 ± 0.586		0.827 ± 0.135	
**Age**					
< 50	32	3.042 ± 0.330	0.906	0.869 ± 0.099	0.406
≥ 50	34	3.110 ± 0.456		0.759 ± 0.087	
**Tumor size**					
< 5 cm	27	4.133 ± 0.444	0.001**	1.073 ± 0.100	0.001**
≥ 5 cm	39	2.346 ± 0.320		0.633 ± 0.075	
**WHO grade**					
I	3	5.353 ± 0.214	0.001**	1.423 ± 0.054	0.003**
II	17	4.215 ± 0.403		1.034 ± 0.075	
III	16	3.503 ± 0.652		0.895 ± 0.119	
IV	30	1.977 ± 0.368		0.583 ± 0.106	
**Extent of resection**					
< 95%	17	3.046 ± 0.862	0.949	0.720 ± 0.174	0.409
≥ 95%	49	3.088 ± 0.243		0.845 ± 0.065	
**KPS score**					
< 80	32	3.073 ± 0.502	0.989	0.706 ± 0.074	0.114
≥ 80	34	3.081 ± 0.285		0.914 ± 0.105	

We also identified the expression levels of miR-30c in the presurgery plasma of patients with gliomas and healthy volunteers. The expression level of miR-30c in the glioma patients was 0.813 ± 0.066, and that in the healthy volunteers was 2.403 ± 0.136. The difference was statistically significant (*P* < 0.01) (Fig. 1B). As illustrated in Table 1, the expression level of miR-30c in the plasma of the glioma patients was closely related to the WHO grade and tumor size (*P* < 0.01) but not to the sex, age, extent of resection, or KPS scores of the patients (*P* > 0.05). The expression levels of miR-30c in tumor tissue were positively correlated with the levels found in plasma according to the Pearson correlation test (r = 0.868, *P* = 0.002) (Fig. 1C). The expression levels of miR-30c in tissues and plasma were consistent.

To discern whether miR-30c can be used to determine the prognosis of gliomas in patients, we determined the expression levels of miR-30c in paraffin-embedded glioma tissues, and we analyzed the correlation between the expression level of miR-30c, progression-free survival (PFS), and overall survival (OS) using Kaplan‒Meier curves. Figure 1D,E shows that the PFS and OS of patients with low expression levels of miR-30c were significantly shorter than those of patients with higher expression levels of miR-30c. Thus, it can be inferred that miR-30c could be used to predict the prognosis of patients with gliomas.

### miR-30c inhibits EMT and the proliferation, migration, and invasion of glioma cells

**Figure 2:**
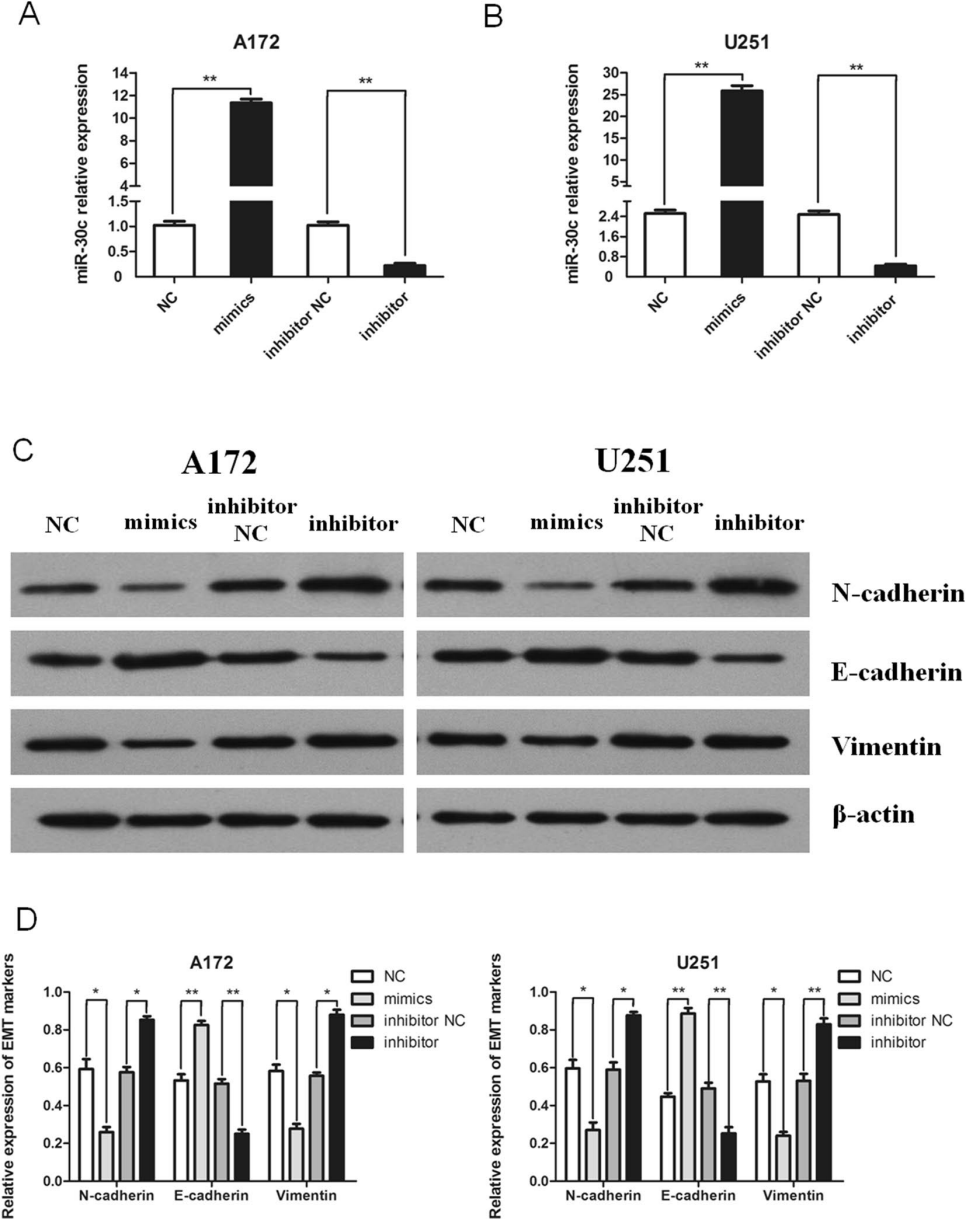
miR-30c inhibits the EMT process of glioma cells. (**A**, **B**) qRT‒PCR was used to verify the change in miR-30c expression levels in A172 and U251 cells after transfection with the mimics and the inhibitor (**P < 0.01). (**C**, **D**) The EMT process of glioma cells was changed after the transfection of the mimics and the inhibitor (*P < 0.05, **P < 0.01).

We then transfected mimics or a negative control (NC) or an inhibitor or an inhibitor NC into A172 and U251 glioma cells. The expression level of miR-30c in the mimics group was significantly higher than that in the NC group (*P* < 0.01). The expression level of miR-30c in the inhibitor group was significantly lower than that in the inhibitor NC group (*P* < 0.01) (Fig. 2A,B).

In the Western blot experiment, the protein expression level of N-cadherin and Vimentin in the mimics group was lower than that in the NC group (*P* < 0.05), and the protein expression level of E-cadherin in the mimics group was higher than that in the NC group (*P* < 0.01). Regarding the inhibitor group, the protein quantity of N-cadherin and Vimentin was higher than that of the inhibitor NC group (*P* < 0.05), and the protein expression of E-cadherin was lower than that in the inhibitor NC group (*P* < 0.01) (Fig. 2C,D). E-cadherin, N-cadherin, and Vimentin are marker genes for the process of EMT. Therefore, we are confident that miR-30c can inhibit the EMT process in glioma cells.

**Figure 3:**
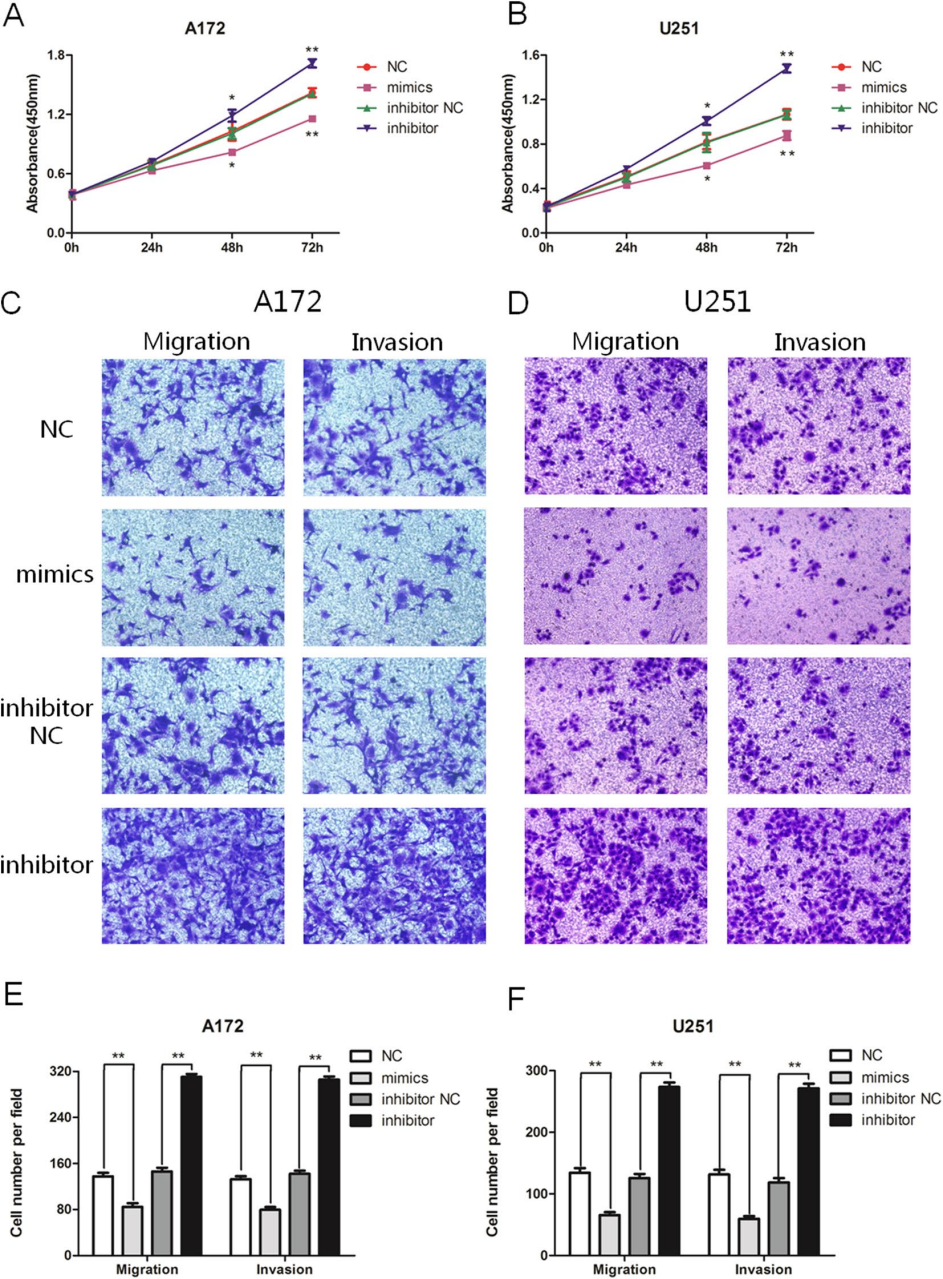
miR-30c reduces the proliferation, migration, and invasion of glioma cells. (**A**, **B**) CCK8 assay revealed that miR-30c could impact the proliferation of A172 and U251 cells (*P < 0.05, **P < 0.01). (**C**, **E**) The migration and invasion of the mimics and inhibitor groups were significantly changed in A172 cells (**P < 0.01). (**D**, **F**) The migration and invasion of U251 cells were significantly changed after transfection with the mimics and the inhibitor (**P < 0.01).

In the cell proliferation experiment, as shown in Fig. 3A,B, the proliferation of glioma cells in the mimics group was less than that in the NC group (*P* < 0.05, T = 48 h; *P* < 0.01, T = 72 h). The cell proliferation of the inhibitor group was greater than that of the inhibitor NC group (*P* < 0.05, T = 48 h; *P* < 0.01, T = 72 h). Therefore, we have determined that miR-30c can potentially inhibit the proliferation of glioma cells.

As shown in Fig. 3C–F, the migration and invasion of A172 and U251 cells transfected with mimics were significantly less than those in the NC group (*P* < 0.01), whereas the migration and invasion of A172 and U251 cells transfected with the inhibitor were significantly greater than those in the inhibitor NC group (*P* < 0.01). Our results suggested that miR-30c has the potential to inhibit the migration and invasion of glioma cells.

### Notch1 is a direct target gene of miR-30c in gliomas

**Figure 4:**
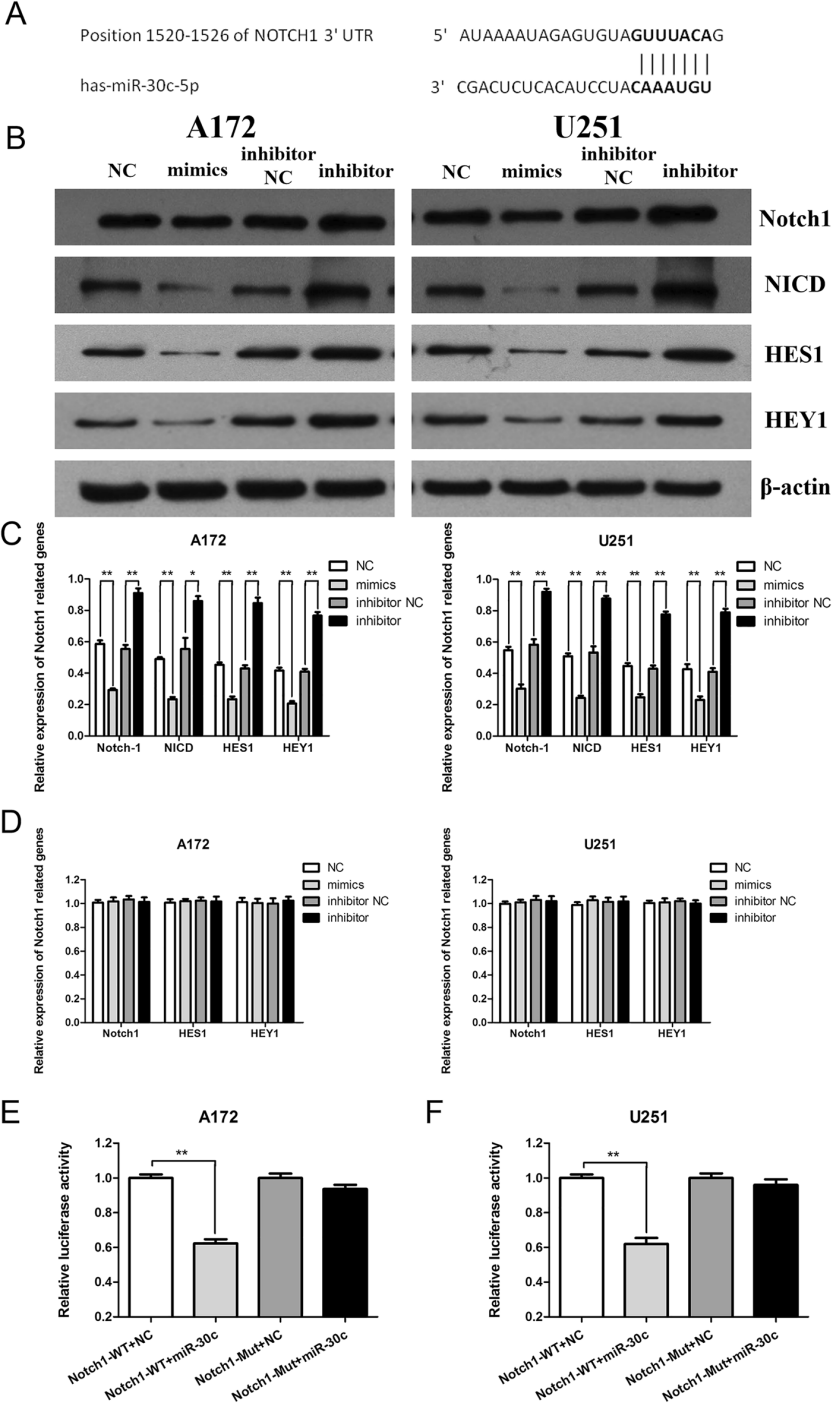
Notch1 is the direct target gene of miR-30c in gliomas. (**A**) The 3′-UTR of Notch1 binds to miR-30c. (**B**, **C**) Changes in the Notch1, NICD, HES1 and HEY1 proteins after transfection with the mimics and the inhibitor (*P < 0.05, **P < 0.01). (**D**) There was no alteration in Notch1, HES1 and HEY1 mRNAs after the transfection of the mimics and the inhibitor. (**E**, **F**) Dual luciferase assay of two kinds of glioma cells (**P < 0.01).

According to the TargetScan, miRWalk, and miRanda databases, we predicted that Notch1 might be the direct target gene of miR-30c, and its action site is shown in Fig. 4A. When mimics were transfected into A172 and U251 cells, the expression levels of the Notch1 protein in the mimics group were significantly lower than those in the NC group (*P* < 0.01). The expression level of NICD protein, which indicates the activation of Notch1 in the mimics group, was also significantly lower than that in the NC group, and the expression levels of downstream target genes of Notch1, such as HES1 and HEY1 proteins, in the mimics group were also significantly lower than those in the NC group (*P* < 0.01) (Fig. 4B,C). However, there was no significant change in Notch1, HES1 and HEY1 mRNA levels (Fig. 4D). When the inhibitor was transfected into glioma cells, the expression levels of the Notch1, NICD, HES1 and HEY1 proteins in the inhibitor group were significantly higher than those in the inhibitor NC group (*P* < 0.05) (Fig. 4B,C). There was no significant change in Notch1, HES1 and HEY1 mRNA levels (Fig. 4D). In the dual luciferase experiment, as shown in Fig. 4E,F, miR-30c reduced the luciferase activity of Notch1-WT in A172 and U251 cells, but it had no effect on Notch1-Mut. Our findings suggest that Notch1 is a direct target gene of miR-30c in gliomas.

### Notch1 can promote EMT and the proliferation, migration, and invasion of gliomas

**Figure 5:**
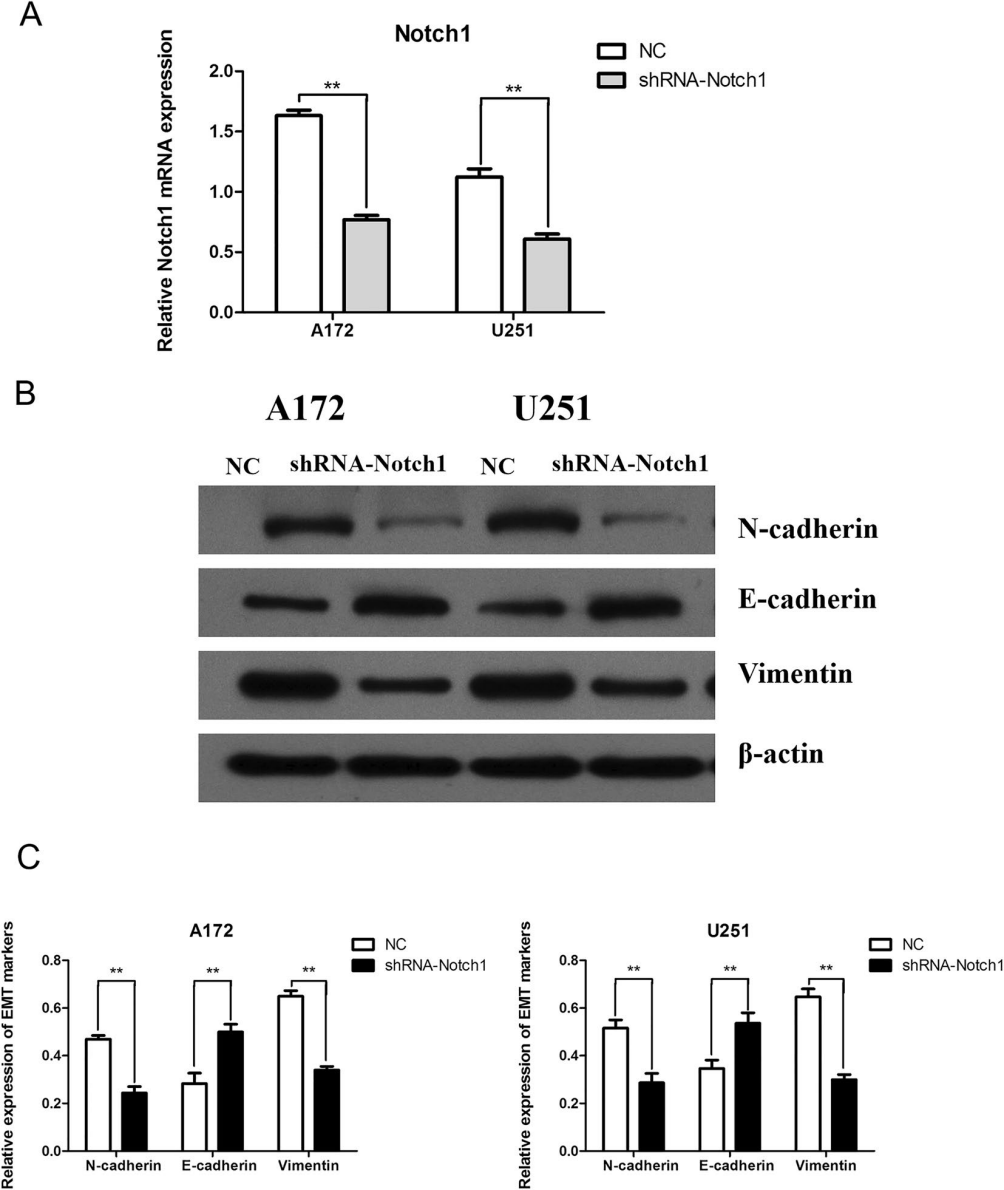
Notch1 can promote the EMT process of gliomas. (**A**) The changes in Notch1 mRNA after shRNA-Notch1 transfection were verified by qRT‒PCR (**P < 0.01). (**B**, **C**) Changes in EMT-related proteins after shRNA-Notch1 transfection (**P < 0.01).

**Figure 6:**
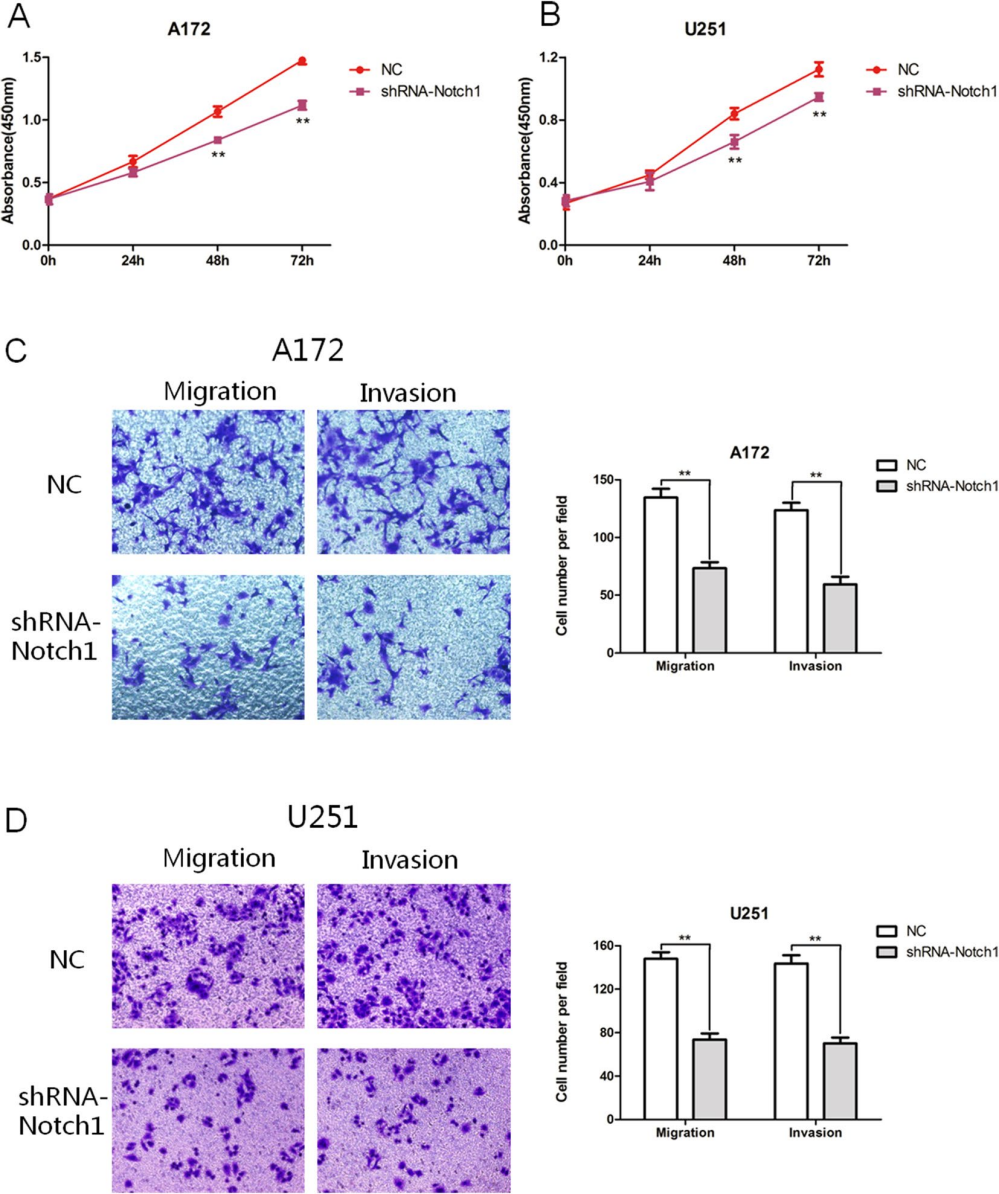
Notch1 can promote the proliferation, invasion, and migration of gliomas. (**A**, **B**) CCK8 assay showed that Notch1 could impact the proliferation of A172 and U251 cells (**P < 0.01). (**C**, **D**) Notch1 changed the cell migration and invasion abilities (**P < 0.01).

shRNA can hinder the expression of Notch1 in glioma cells. In Fig. 5A, qRT-PCR detection revealed that the expression level of Notch1 mRNA following shRNA-Notch1 transfection was notably less than that in the NC group (*P* < 0.01), which provided important information for subsequent experiments. As shown in Fig. 5B,C, the protein levels of N-cadherin and Vimentin in the shRNA-Notch1 group were lower than those in the NC group (*P* < 0.01), and the protein levels of E-cadherin in the shRNA-Notch1 group were higher than those in the NC group (*P* < 0.01). Figure 6A,B shows that inhibition of Notch1 expression can reduce the proliferation of A172 and U251 cells (*P* < 0.01, T = 48h; *P *< 0.01, T = 72h) and reduce the expression of Notch1 in glioma cells, which can significantly curtail cell migration and invasion (*P* < 0.01) (Fig. 6C,D). Therefore, Notch1 can promote EMT and the proliferation, migration, and invasion of gliomas.

### miR-30c inhibited EMT and the proliferation, migration, and invasion of gliomas by reducing Notch1 expression

**Figure 7:**
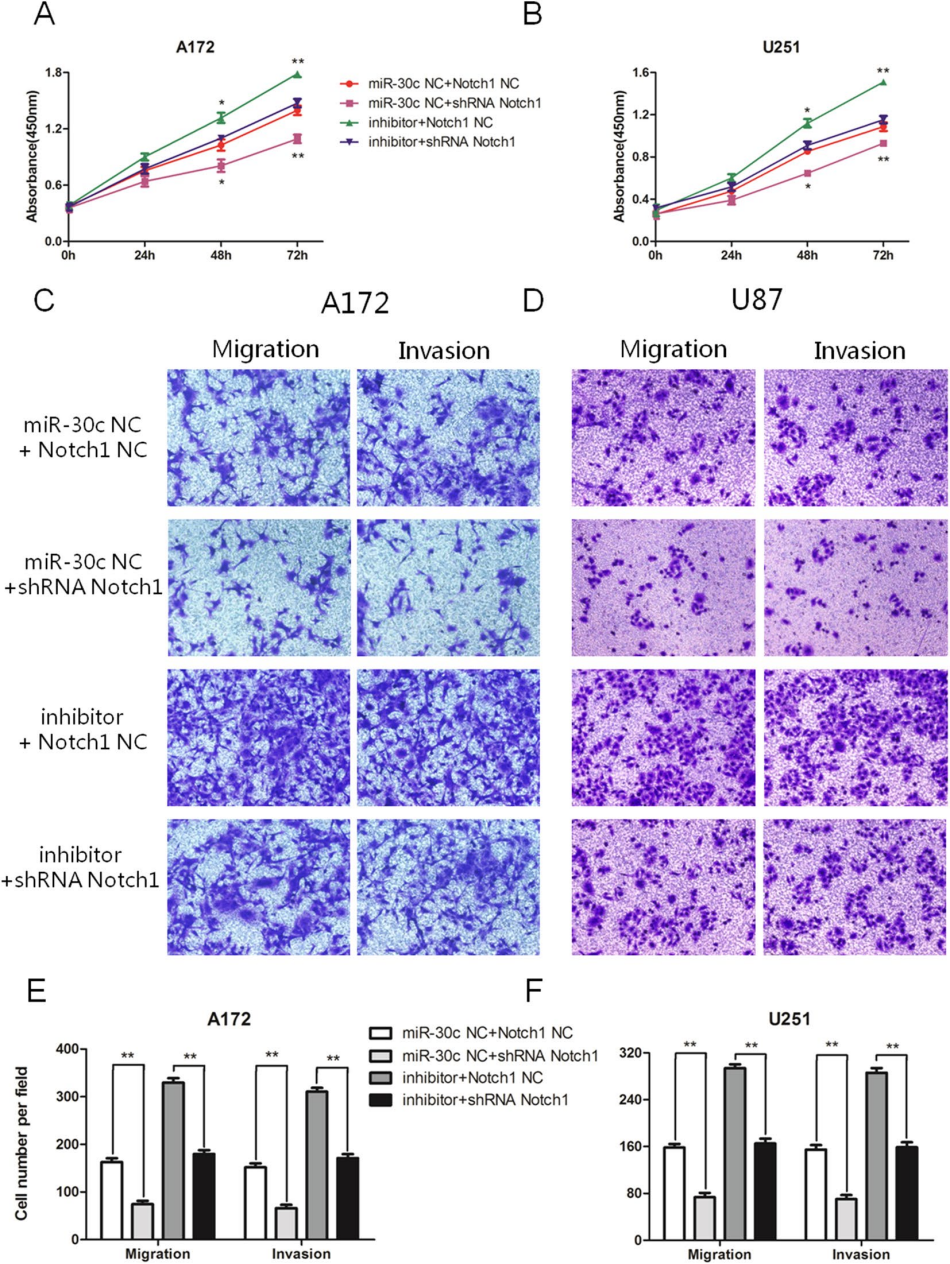
miR-30c hindered proliferation, migration, and invasion by downregulating the expression levels of Notch1. (**A**, **B**) The miR-30c inhibitor facilitated an increase in the proliferation of glioma cells, while shRNA-Notch1 attenuated this change (*P < 0.05, **P < 0.01). (**C**, **D**, **E**, **F**) shRNA-Notch1 attenuated the enhancement of migration and invasion of glioma cells by the miR-30c inhibitor (**P < 0.01).

**Figure 8:**
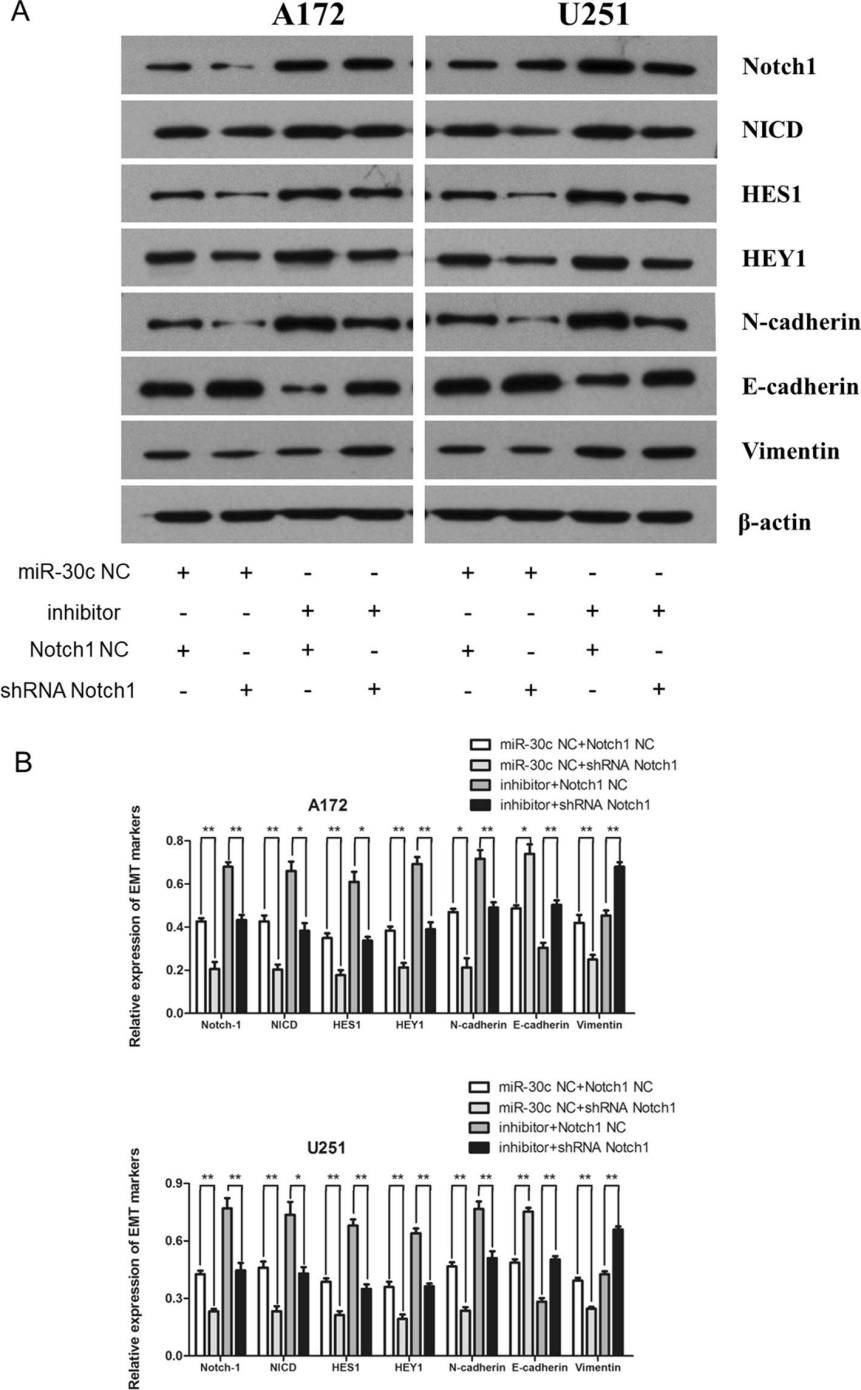
The miR-30c-mediated EMT depended on Notch1. (**A**, **B**) shRNA-Notch1 attenuated the effect of the miR-30c inhibitor on the Notch1, NICD, HES1 and HEY1 proteins of A172 and U251 glioma cells. The miR-30c inhibitor triggered an increase in N-cadherin and a decrease in E-cadherin, while cotransfection with shRNA-Notch1 partially eliminated these effects. However, the expression of Vimentin was not affected (*P < 0.05, **P < 0.01).

To more fully discern the mechanism of miR-30c in gliomas and to clarify that miR-30c can inhibit the EMT process of gliomas by reducing the expression levels of Notch1, we implemented a cotransfection experiment. By cotransfecting the miR-30c inhibitor and shRNA-Notch1 into A172 and U251 cells, we revealed that the miR-30c inhibitor could enhance the proliferation of glioma cells, while shRNA-Notch1 could attenuate this change (*P* < 0.05, T = 48h; *P* < 0.01, T = 72h) (Fig. 7A,B). Moreover, we discovered that shRNA-Notch1 can attenuate the effect of the miR-30c inhibitor on the migration and invasion of A172 and U251 glioma cells (Fig. 7C–F). The miR-30c inhibitor enhanced the Notch1, NICD, HES1 and HEY1 proteins in A172 and U251 glioma cells, and shRNA-Notch1 weakened this enhancement (Fig. 8A,B). The expression of the key factors N-cadherin increased, and the expression of E-cadherin decreased in the EMT process induced by the inhibitor. However, cotransfection of shRNA-Notch1 attenuated the changes in key gene proteins in EMT. But the expression of Vimentin was not affected in the cotransfection experiments (Fig. 8A,B). Our results suggest that miR-30c could inhibit EMT and the proliferation, migration, and invasion of glioma cells by directly acting on Notch1.

## Discussion

miRNA target genes are large in number and wide in variety and are involved in multiple cellular signal transduction pathways. They not only create a complex signal regulatory network but also serve an assortment of biological purposes^28–30^. In recent years, research has indicated that miR-30c is actively involved in the regulation of malignant tumor signaling pathways and that it acts as a tumor suppressor gene in the majority of malignant tumors, such as renal cell carcinoma, colorectal cancer, gastric cancer, and breast cancer^31–34^. Sox9 is a high-mobility group box transcription factor that plays critical roles during embryogenesis, differentiation, tumor initiation, invasion and stem cell self-renewal^35,36^. Sox9 is critical for tumorigenesis and is overexpressed in gliomas^37,38^. In glioblastoma, miR-30c can impede the proliferation, migration and invasion of tumor cells by affecting Sox9^39^. The latest research showed that Notch1 activation induced strong Sox9 expression^40^. Therefore, Notch1 is more important in the pathogenesis of gliomas. Apoptosis is triggered by Bcl-2 superfamily members. miR-30c targets Bcl-2 and activates the caspase pathway in glioma cells^41^. Glioma cell proliferation is inhibited, and cell apoptosis is promoted through the suppression of Notch1^42^. Therefore, if the apoptosis of tumor cells is decreased in gliomas, the expression of Notch1 needs to be inhibited first. Based on the important role of Notch1, we were eager to determine which miRNA mainly regulates it in gliomas. Our study explored all levels of gliomas and discovered that even in low-grade gliomas, the expression levels of miR-30c decreased to a significant extent and that those with a higher grade had lower expression levels of miR-30c. The expression level of miR-30c in glioma tissues and preoperative plasma was closely related to the WHO grade and tumor size, and the expression level of miR-30c in tissues and plasma remained consistent. Moreover, in paraffin sections, with lower miR-30c levels, we noted reduced PFS and OS, and such information can be used to predict the prognosis of glioma patients.

miR-30c is closely related to a variety of cell biological functions. Unlimited cell proliferation is an important biological feature of malignant tumor cells. In this experiment, miR-30c was discovered to hinder the proliferation of glioma cells. EMT is defined as a process by which epithelial tumor cells lose their adhesive ability and obtain mesenchymal cell migration ability to promote metastasis and drug resistance. Our research indicated that increasing the expression level of miR-30c can reduce the protein expression of N-cadherin and Vimentin, just as it can increase the protein expression of E-cadherin, thereby inhibiting the EMT processes of glioma cells. Additionally, tumor metastasis is a multistage and multistep orderly process, and tumor migration and invasion are the premise of tumor metastasis. We determined that miR-30c can inhibit the migration and invasion of glioma cells.

Based on a database analysis, we predicted that Notch1, which functions as an important element in glioma, may be the direct target gene of miR-30c. By increasing the expression level of miR-30c, the expression level of Notch1 protein decreased; conversely, when the expression level of miR-30c was inhibited, the expression of Notch-1 protein increased. However, there was no effect on Notch1 mRNA. In the dual luciferase assay, miR-30c reduced the levels of luciferase activity of Notch1-WT in glioma cells but had no effect on Notch1-Mut. Therefore, we believe that Notch1 is the direct target gene of miR-30c and functions posttranscriptionally.

Notch1 plays an instrumental role in glioma cells, with the functions of enhancing stemness and tumorigenicity^43,44^, promoting tumor growth^45^ and EMT^46^, and increasing chemotherapy drug resistance^47^. In this study, we used shRNA to inhibit the expression levels of Notch1 in glioma cells, and we revealed that EMT (N-cadherin decreased, E-cadherin increased) and the proliferation, migration, and invasion of cancer cells were significantly decreased. This further confirmed the carcinogenic effect of Notch1. Afterward, we cotransfected the miR-30c inhibitor and shRNA-Notch1 into two glioma cell lines. shRNA-Notch1 attenuated the enhancement effect of the inhibitor on EMT and the proliferation, migration, and invasion of glioma cells, thus confirming that miR-30c regulates these processes in glioma cells posttranscriptionally by targeting Notch1. The expression of Vimentin was not affected, we considered that there may be the following two reasons: First, transcription factors such as SNAI1, SNAI2, TWIST1, and ZEB-1 play the important roles in mesenchymal differentiation^48,49^. The depletion of TWIST1/SNAI1 resulted in reduced Bcl-w-induced Vimentin expression and invasion^50^. Second, we speculate that the occurrence of this situation may also be related to Notch1 expression. This may be related to the heterogeneity of tumor cells.

In conclusion, the expression level of miR-30c in glioma tissue and preoperative plasma was decreased, and the low expression level was closely tied to the WHO grade, tumor size, and prognosis. miR-30c can inhibit EMT and the proliferation, migration, and invasion of glioma cells. miR-30c acts as a tumor suppressor gene by targeting Notch1 at the posttranscriptional level. Therefore, miR-30c may act as a marker for tumor diagnosis, treatment, and prognosis.

## Materials and methods

### Clinical specimens

Sixty-six patients with brain glioma who were first diagnosed at Taian Central Hospital (Taian, Shandong, China) from June 2018 to May 2020 were selected, and 2 ml of peripheral venous plasma was collected from each patient prior to surgery. The other 9 normal, nonglioma brain tissue samples were obtained from patients after decompression surgery to treat traumatic brain injury, which was confirmed as normal brain tissue by postoperative pathology. Control plasma samples were collected from 9 healthy volunteers.

Brain glioma paraffin specimens from 72 patients were collected from Taian Central Hospital from June 2013 to May 2018. Eight of the 72 patients were lost during the follow-up stage, and 64 patients were ultimately assessed. The included patients were diagnosed with glioma by postoperative pathology and were graded according to the WHO classification and the grading standard of nervous system tumors (2016). None of the patients received any form of radiotherapy, chemotherapy, targeted therapy, immunotherapy, or any other anticancer treatments prior to operation, and each study participant signed the informed consent form before sampling. The ethics committee of the Taian Central Hospital approved this study. All methods were performed in accordance with the relevant guidelines and regulations.

### Cell culture

U251 cells were purchased from Shanghai Cell Bank at the Chinese Academy of Medical Sciences (Shanghai, China), and A172 cells were from the Central Laboratory of Taian Central Hospital. Both types of cells were cultured in DMEM (GIBCO, Gaithersburg, MD, USA) containing 10% fetal bovine serum (FBS, GIBCO) and 0.2% penicillin streptomycin (Invitrogen, Carlsbad, California, USA). The cells were cultured in an incubator at 37 °C with 95% humidity and 5% CO2.

### Real-time quantitative PCR (qRT‒PCR)

Following the instructions of the miRNeasy Mini Kit (Qiagen, Duesseldorf, Nordrhein-Westfalen, Germany), total RNA was extracted from glioma tissue, normal brain tissue, and glioma cells. Total RNA was extracted from plasma by using the miRNeasy Serum/Plasma Kit (Qiagen), and FFPE tissue samples were extracted using the miRNeasy FFPE Kit (Qiagen). The reverse transcription experiment was carried out by using the miScript II RT Kit (Qiagen) to synthesize cDNA. The miScript SYBR Green PCR Kit (Qiagen) was implemented to measure the qRT-PCR of miRNA. Primer sequences were used as follows: miR-30c forward, 5′-TGTGTTTTTATTGTTTTTGTTGTCCCA-3′; miR-30c reverse, 5′-GGGACAGAACAGGTTAATGGGAA-3′; U6-forward, 5′-GCTTCGGCAGCACATATACT- AAAAT-3′; and U6-reverse, 5′-CGCTTCACGAATTTGCGTGTCAT-3′.

The reaction was performed in a LightCycler 480 qRT‒PCR system (Roche molecular systems, Indianapolis, USA) according to the instructions. After amplification, the cycle threshold (CT) of each PCR was calculated by measuring the number of cycles required when the cDNA template reached the set fluorescence intensity by PCR. Three multiple wells were set in each sample, and U6 was used as an internal reference to normalize the target gene. The relative expression of miR-1 was determined according to 2^−∆∆Ct^. TB Green™ *Premix Ex Taq*™ II (Tli RNaseH plus) (Takara, Japan) was used to analyze the PCR of mRNA. β-actin was used as an internal reference. The primer sequences used were as follows:

Notch1 forward, 5′-GCTACAACTGCGT GTGTGTC-3′; Notch1 reverse, 5′-GTTGGTGTC- GCAGTTGGAGC-3′; HES1 forward, 5′-CTGAGCACAGACCCAAGTGT-3′; HES1 reverse, 5′-GAGTGCGCACCTCGGTATTA-3′; HEY1 forward, 5′-GTGCGGACGAGAATGGAAAC-3′; HEY1 reverse, 5′-TTGCTCCATTACCTGCTTCTCA-3′; β-actin forward, 5′-CAAAGGCCAACAGAGAGAAGAT-3′; and β-actin reverse, 5′-TGAGACACACCATCACCAGAAT-3′. The relative expression of Notch1 was determined according to 2^−∆∆Ct^.

### Cell transfection

In six-well plates, as glioma cells reached 70–80% confluence, transfection was executed. Mimics that increased the expression levels of miR-30c, negative control (NC), inhibitor suppressed the expression levels of miR-30c or inhibitor negative control (inhibitor NC), and shRNA inhibiting Notch1 expression or shRNA negative control (shRNA NC) (Gemma, Shanghai, China) were diluted by using Opti-MEM™ to a suitable concentration. Next, Lipofectamine™ 3000 transfection reagent (Invitrogen) was added. After being incubated for 10–15 min at room temperature, the mixture was added to the well plates. After transfection for 24–72 h, the cells were collected and saved for later experiments.

### Cell proliferation, migration, and invasion experiments

The cell proliferation of miR-30c was detected by using the CCK-8 (Cell Counting Kit-8, Beyotime, Shanghai, China) assay. Logarithmic growth phase glioma cells were transferred into 96-well plates, with approximately 2 × 10^3^ cells per well. At 0, 24, 48, and 72 h posttransfection, 10 μl of CCK-8 was added to each well. After adding CCK-8, the cells were placed in an incubator at 37 °C with 5% CO2. After 2 h, the absorbance was detected at 450 nm by using a microplate reader.

The cell migration experiment was carried out in a Transwell chamber with 24-pore plates and 8-µm holes. DMEM containing 20% fetal bovine serum was added to the plates. Next, 1 × 10^5^ transfected glioma cells (200 µl, serum-free medium) were added to the upper chamber. After 24 h, the glioma cells reached the opposite side of the membrane through the pores on the filter membrane and were then fixed with methanol for 15 min and stained with 0.1% crystal violet for 20 min. Five visual fields were selected to count at random. Matrigel (Matrigel/DMEM = 8:1, BD, Franklin, NJ, USA) was added to the upper chamber, with each chamber containing 60 µl. The same method was employed for the cell invasion experiment, and fixation and staining were performed at 48 h.

### Luciferase reporter assay

A172 and U251 glioma cells were placed into 96-well plates. The Notch1 wild-type vector (pGL3-Notch1-3′-UTR-WT) or mutant vector (pGL3-Notch1-3′-UTR-Mut) and miR-30c mimics or NC were cotransfected with Lipofectamine™ 3000 (Invitrogen). At 48 h after transfection, the luciferase activity was measured using the dual luciferase reporter system (Promega, Madison, WI). The ratio of firefly to Renilla luciferase activity was defined as the normalized luciferase activity.

### Western blotting

Cell lysate was used to treat cells and tissues. The protease inhibitor PMSF (Beyotime) was added. The BCA method was used to measure the protein concentration. Proteins were extracted by SDS‒PAGE and then transferred to nitrocellulose membranes. The nitrocellulose membranes were blocked with 5% skimmed milk prepared with TBST and subsequently incubated with the diluted primary antibodies, namely, anti-Notch1 (1:1000), anti-Vimentin (1:1000), anti-N-cadherin (1:1000), anti-E-cadherin (1:1000), anti-NICD (1:1000), and anti-HES1 (1:1000) (Cell Signaling Technology, CST, Boston, USA), anti-HEY1(1:1000) (Abcam, Cambridge, UK) at 4 °C overnight. After TBST was eluted on three occasions, horseradish peroxidase-labeled anti-rabbit or anti-mouse secondary antibodies (CST) were added and incubated on a shaking table for 1 h at room temperature. Then, the target bands were imaged using a chemiluminescence detection system (EMD, Millipore, Billerica, MA, USA) and analyzed quantitatively by using ImageJ software (National Institutes of Health, Bethesda, MD, USA). β-actin (1:5000) was used as an internal reference.

### Statistical analysis

The experimental results are expressed as the means ± SEMs. An independent-samples T test was implemented to detect the differences among glioma tissues and normal tissues after brain trauma decompression, preoperative plasma of glioma patients, and plasma of healthy volunteers. ANOVA was used to evaluate the expression of miR-30c according to various clinicopathological features. Next, Pearson analysis was used to evaluate the connection between miR-30c expression and clinicopathological features. Cytological experiments were then analyzed with a paired-samples T test. A result of *P* < 0.05 was considered statistically significant, and *P* < 0.01 was considered remarkably statistically significant.

## Supplementary Information

Supplementary Information 1.

Supplementary Information 2.

Supplementary Information 3.

Supplementary Information 4.

Supplementary Information 5.

Supplementary Information 6.

Supplementary Information 7.

Supplementary Information 8.

Supplementary Information 9.

Supplementary Information 10.

Supplementary Information 11.

Supplementary Information 12.

Supplementary Information 13.

Supplementary Information 14.
