# Supplementary figures and images for: miR-30c plays diagnostic and prognostic roles and mediates epithelial–mesenchymal transition (EMT) and proliferation of gliomas by affecting Notch1
Source: Sci Rep. 2022 Sep 30;12:16404. doi: 10.1038/s41598-022-19326-x (PMC9525598; doi:10.1038/s41598-022-19326-x)

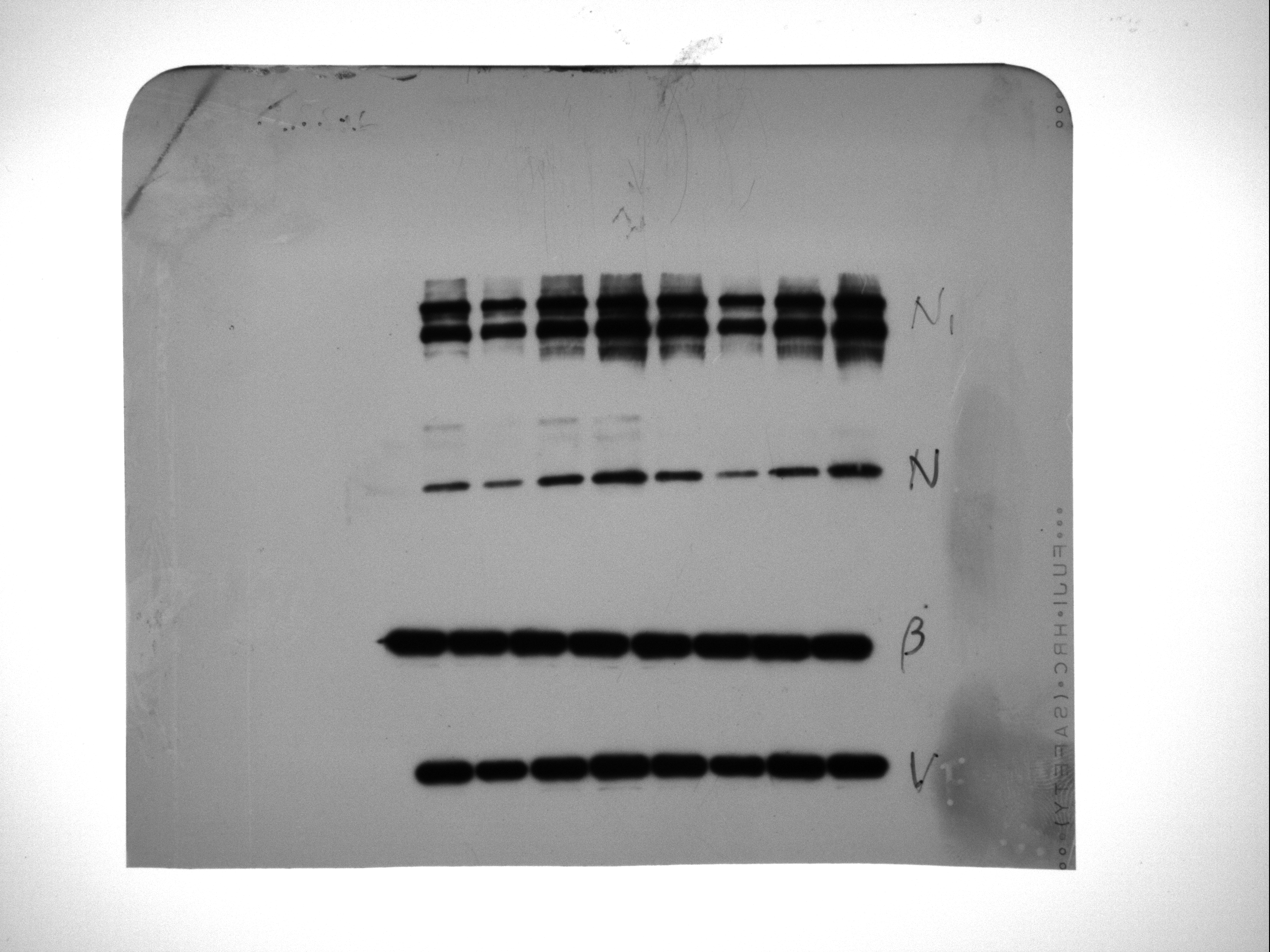

Supplement: Supplementary file 1 — Supplementary Information 1. [file 41598_2022_19326_MOESM1_ESM.jpg]

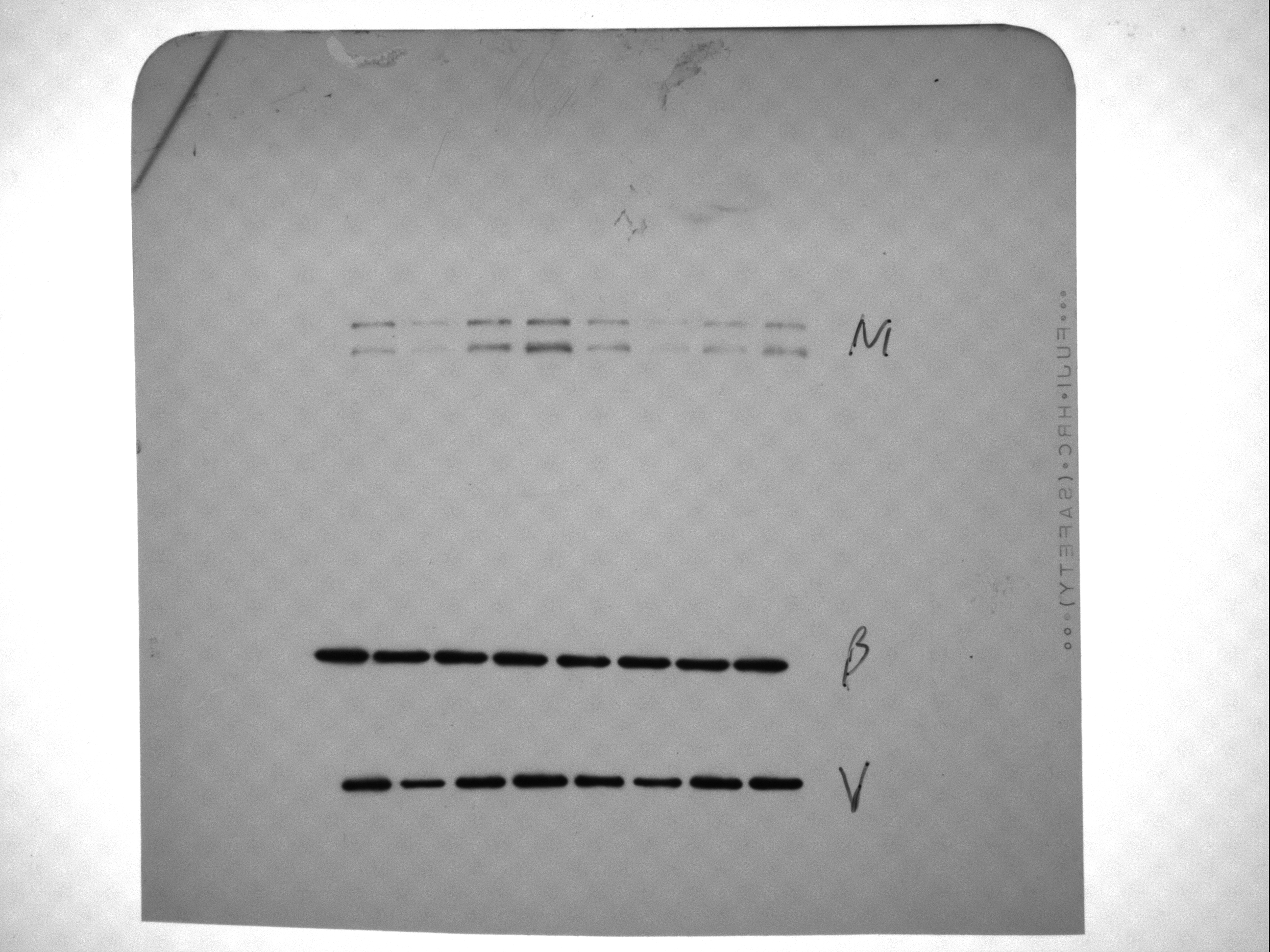

Supplement: Supplementary file 2 — Supplementary Information 2. [file 41598_2022_19326_MOESM2_ESM.jpg]

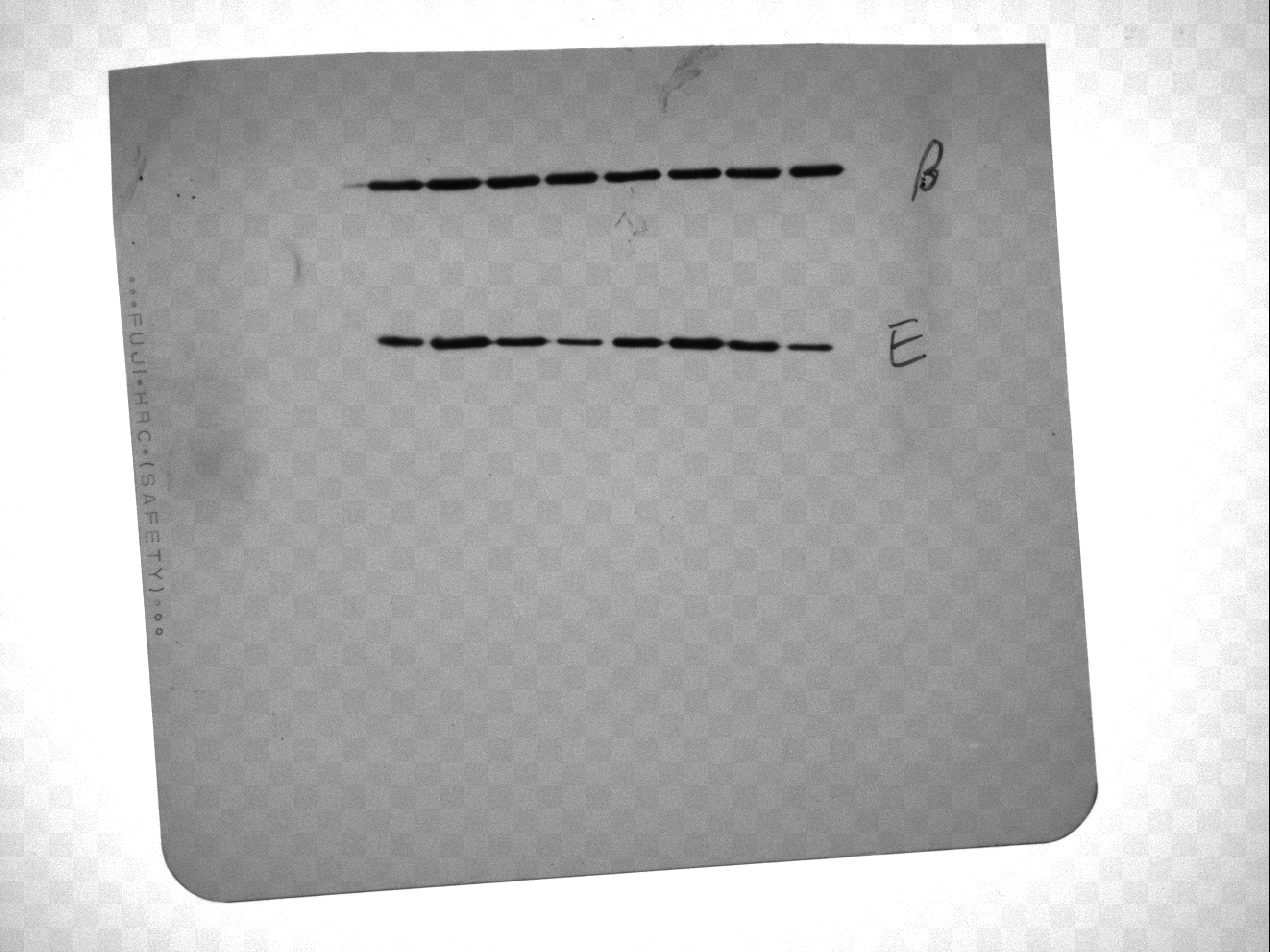

Supplement: Supplementary file 3 — Supplementary Information 3. [file 41598_2022_19326_MOESM3_ESM.jpg]

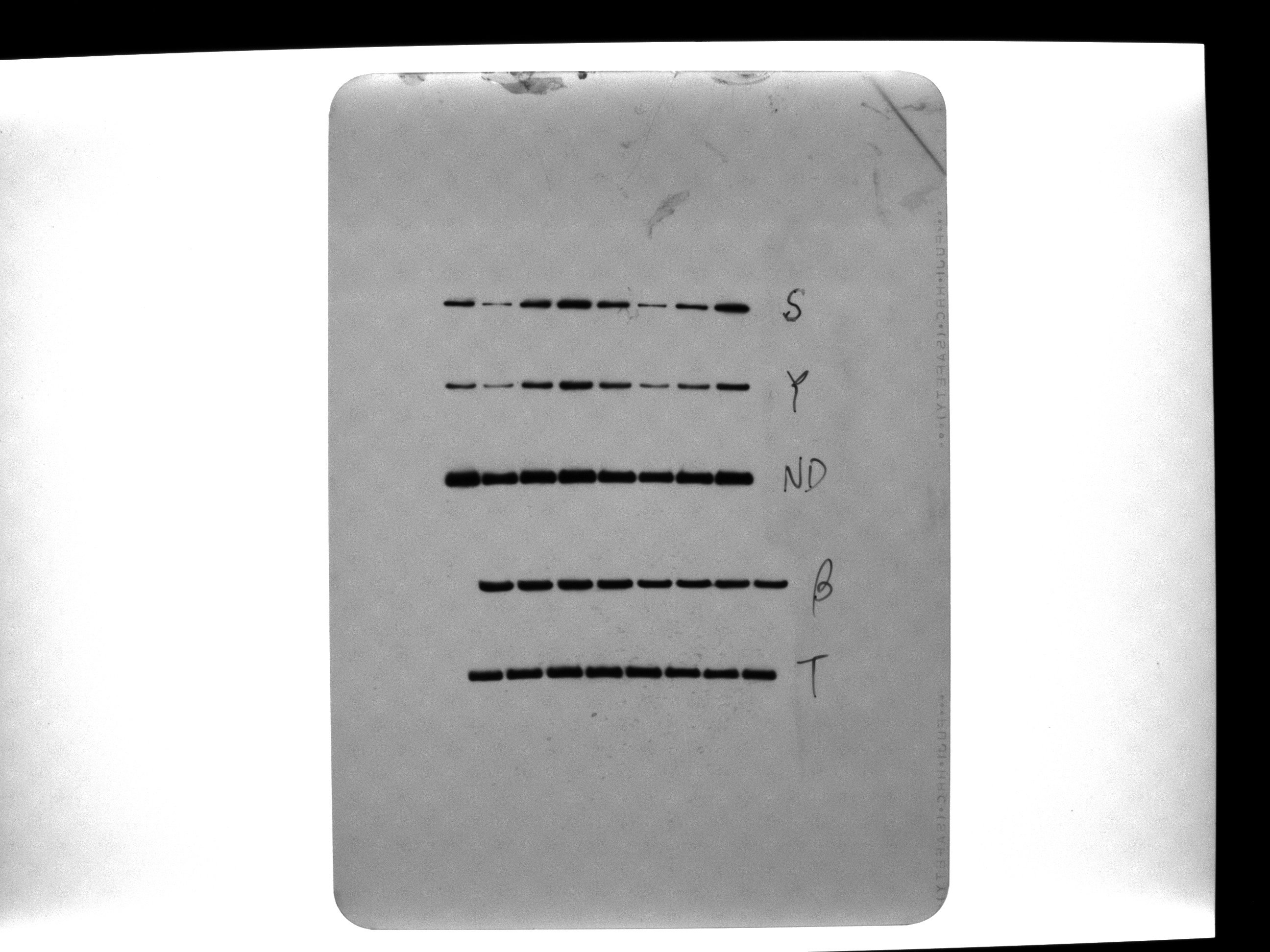

Supplement: Supplementary file 4 — Supplementary Information 4. [file 41598_2022_19326_MOESM4_ESM.jpg]

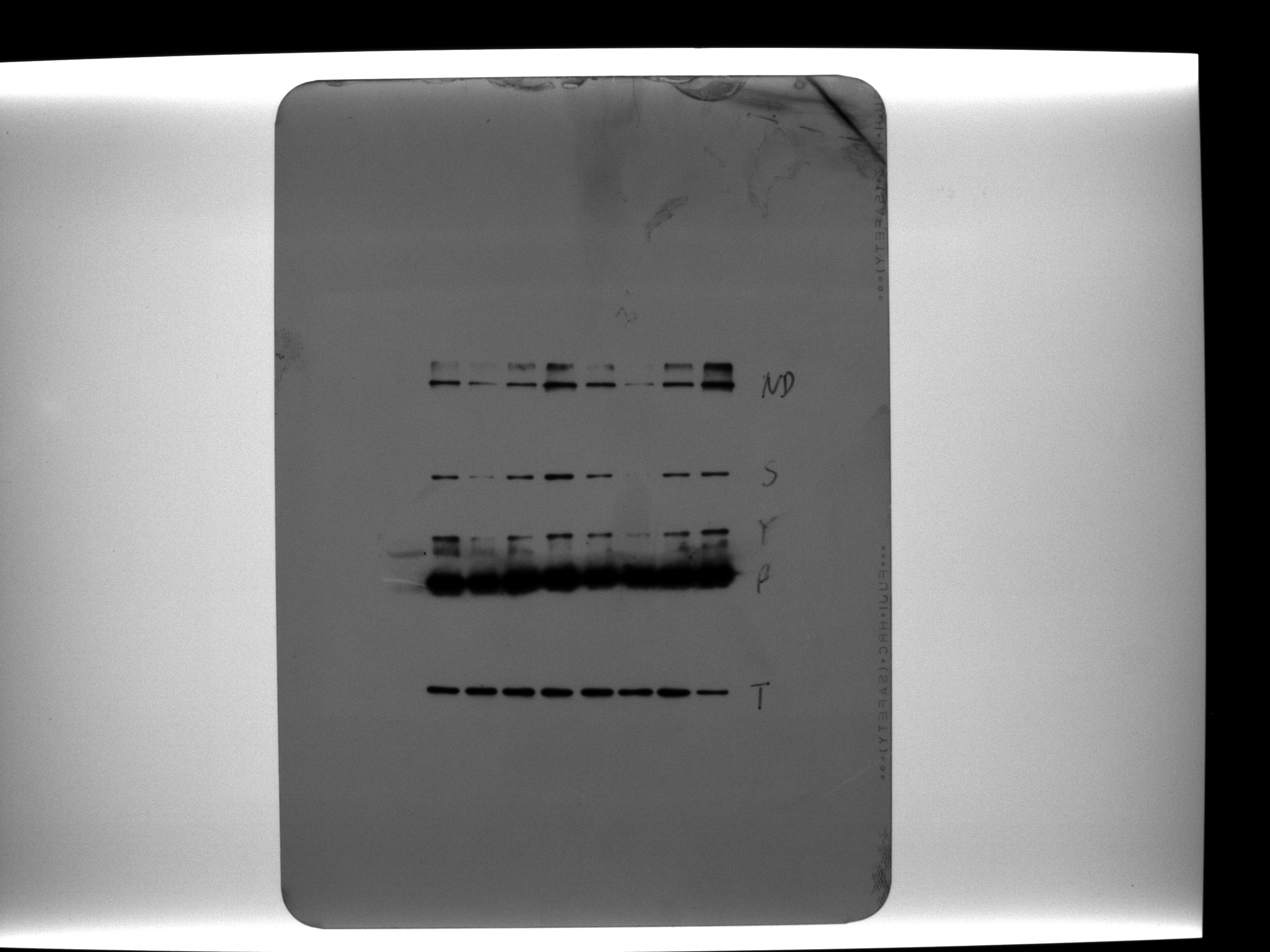

Supplement: Supplementary file 5 — Supplementary Information 5. [file 41598_2022_19326_MOESM5_ESM.jpg]

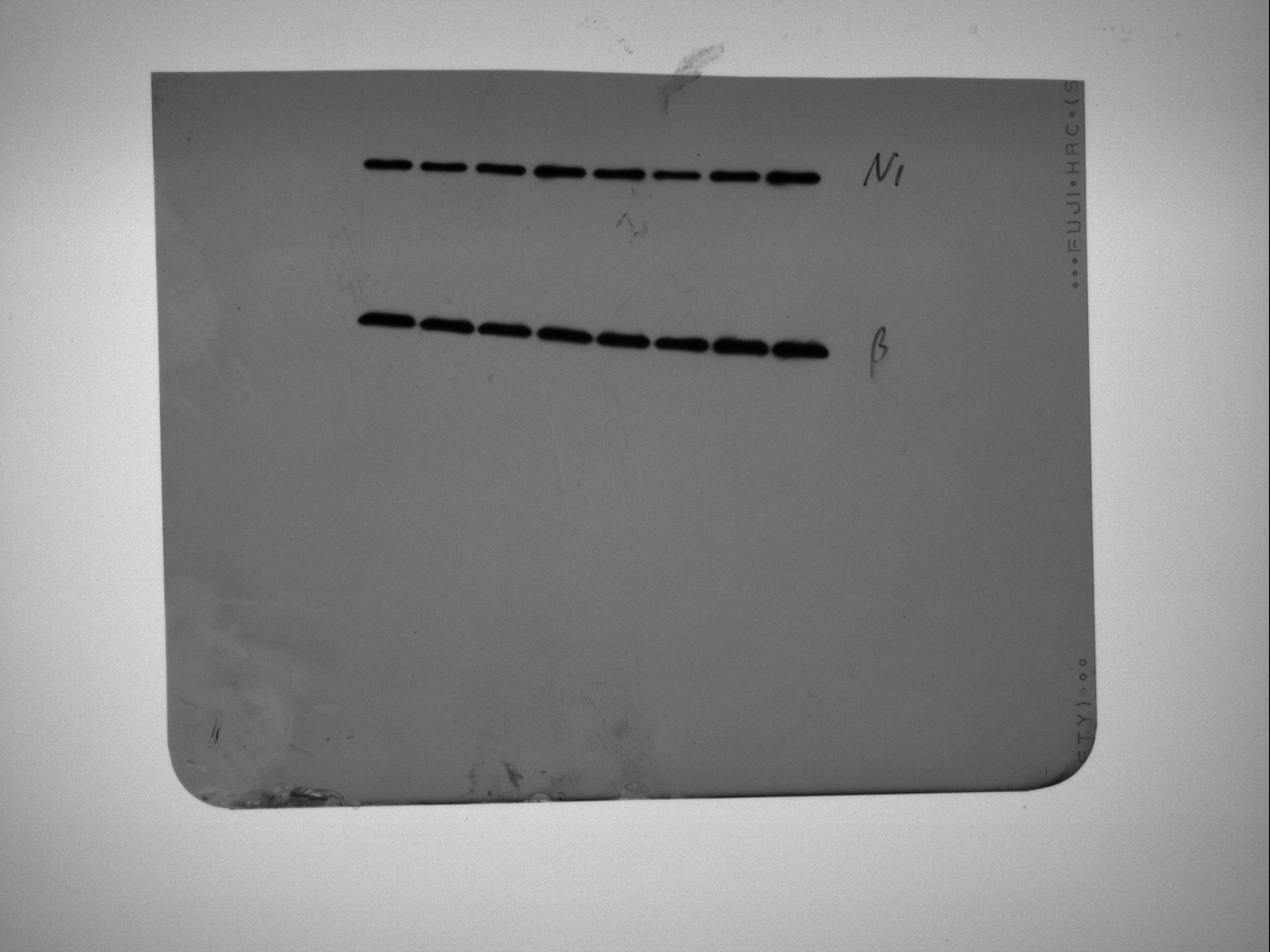

Supplement: Supplementary file 6 — Supplementary Information 6. [file 41598_2022_19326_MOESM6_ESM.jpg]

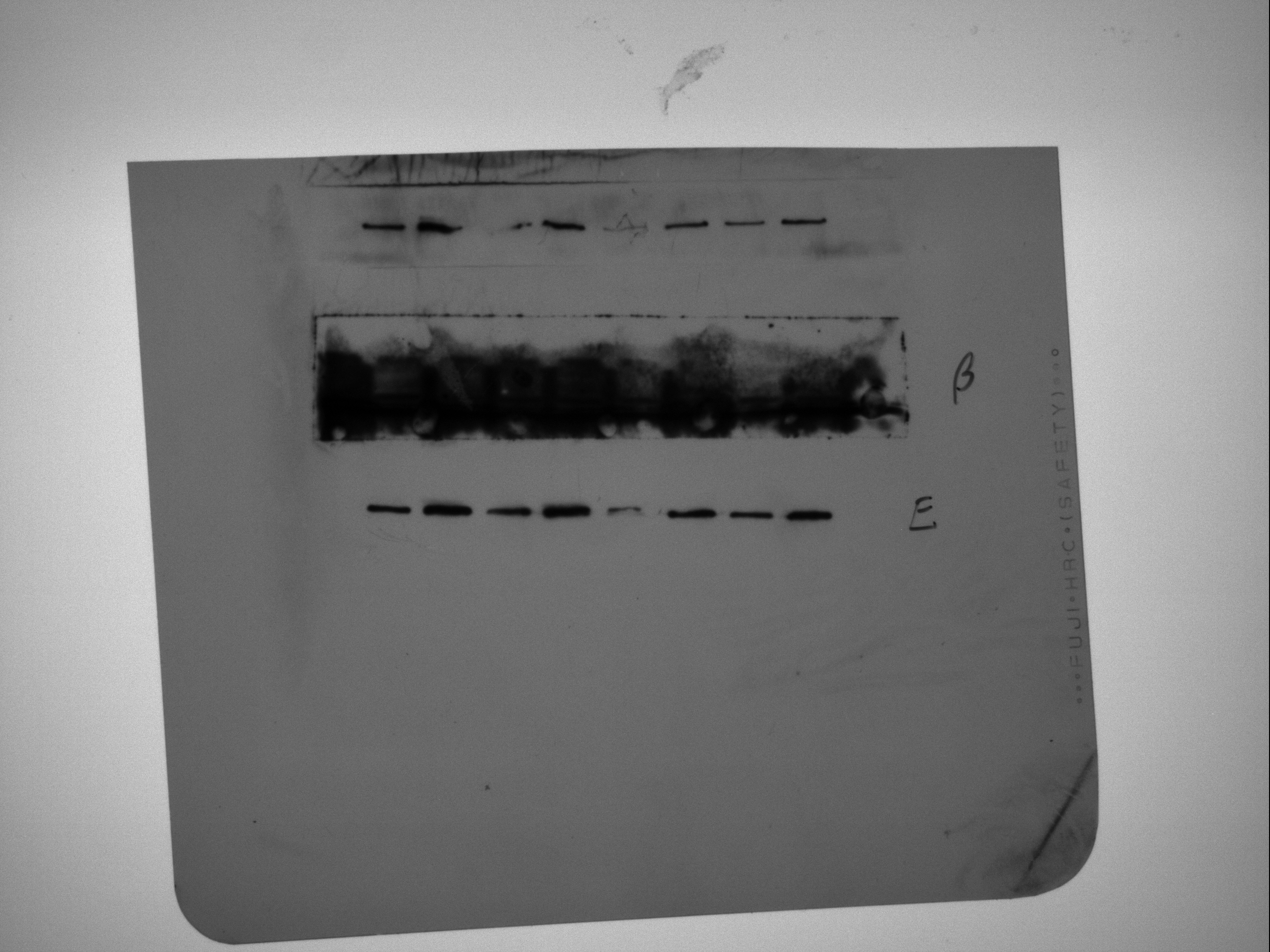

Supplement: Supplementary file 7 — Supplementary Information 7. [file 41598_2022_19326_MOESM7_ESM.jpg]

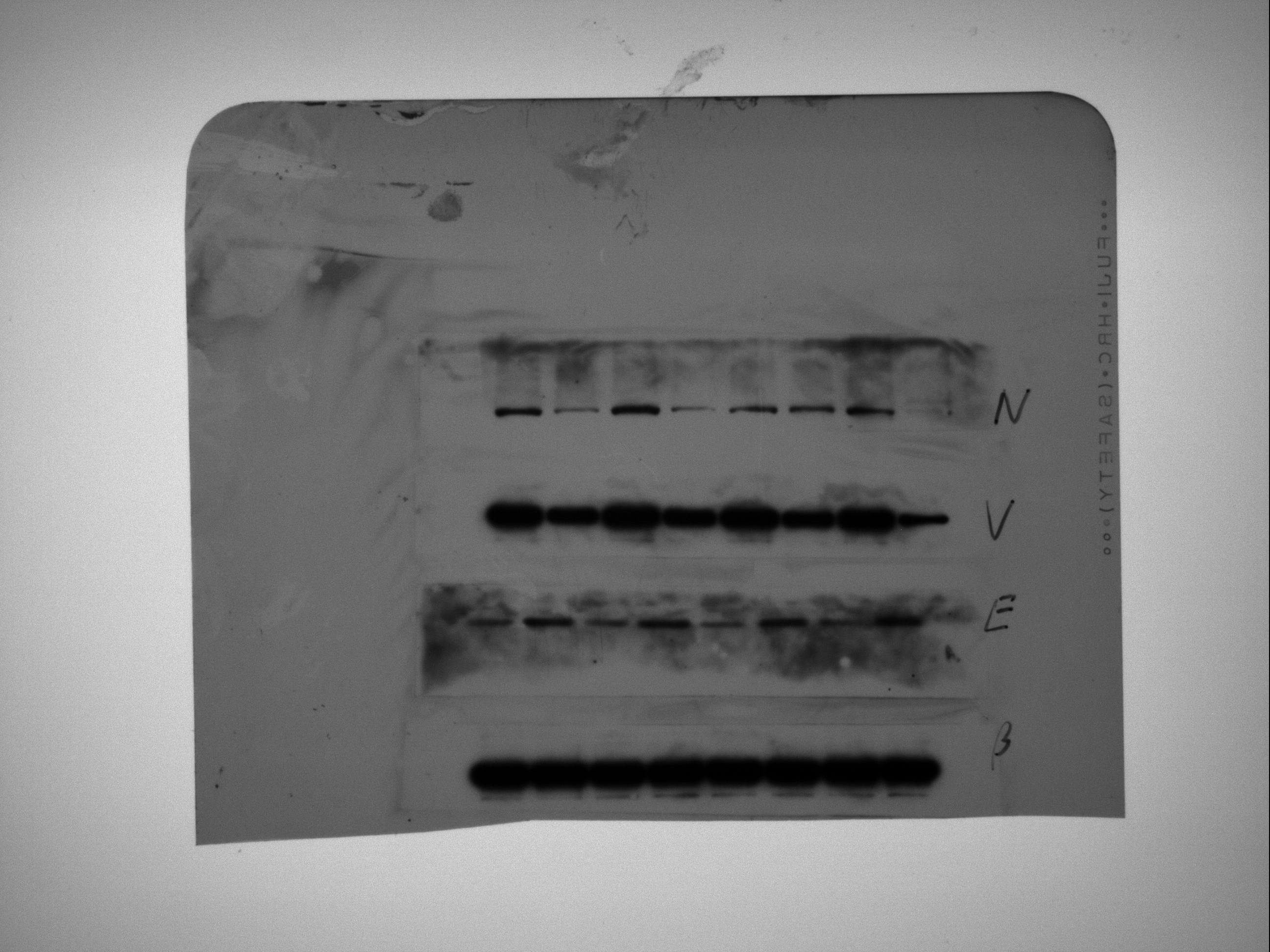

Supplement: Supplementary file 8 — Supplementary Information 8. [file 41598_2022_19326_MOESM8_ESM.jpg]

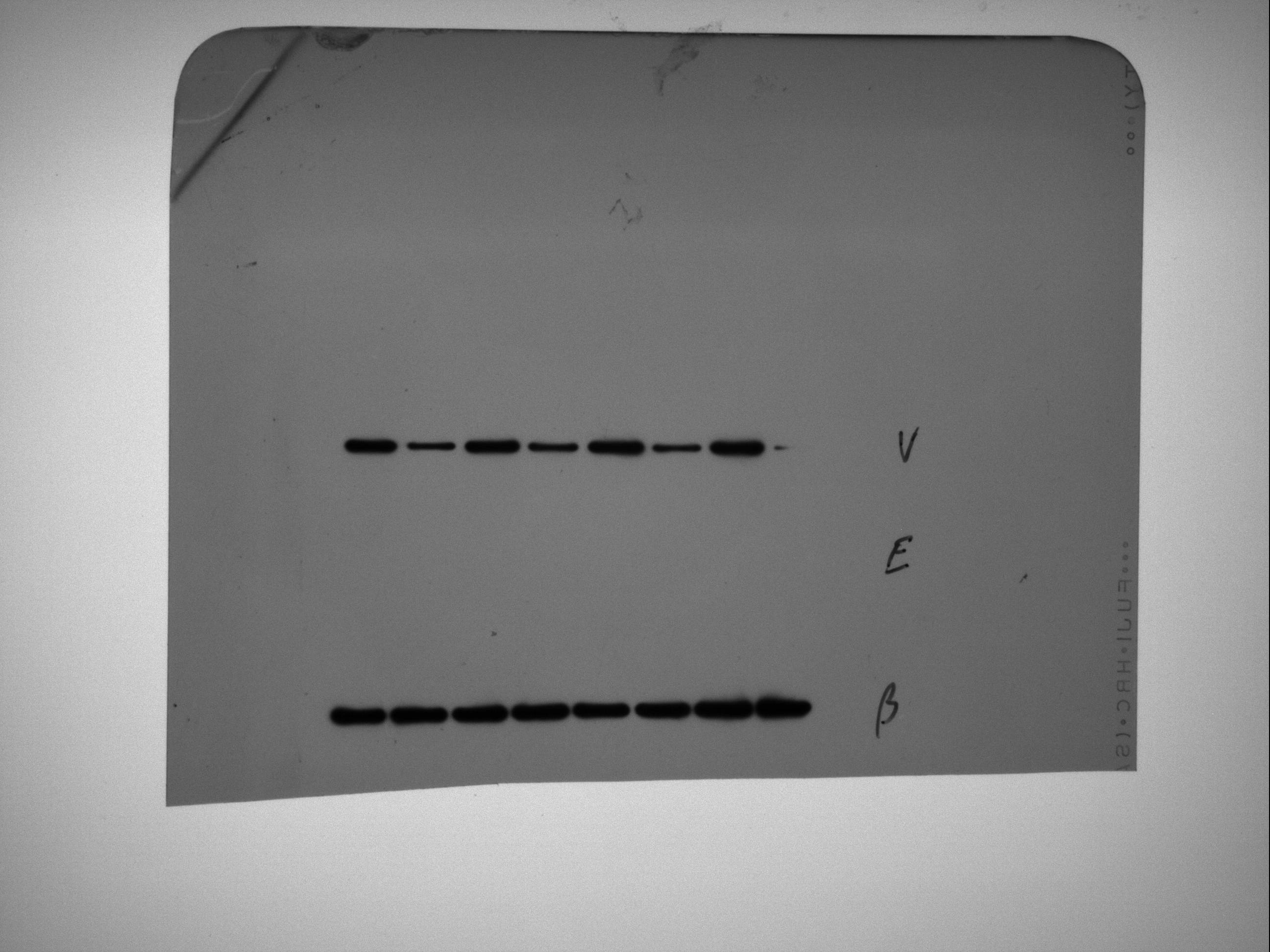

Supplement: Supplementary file 9 — Supplementary Information 9. [file 41598_2022_19326_MOESM9_ESM.jpg]

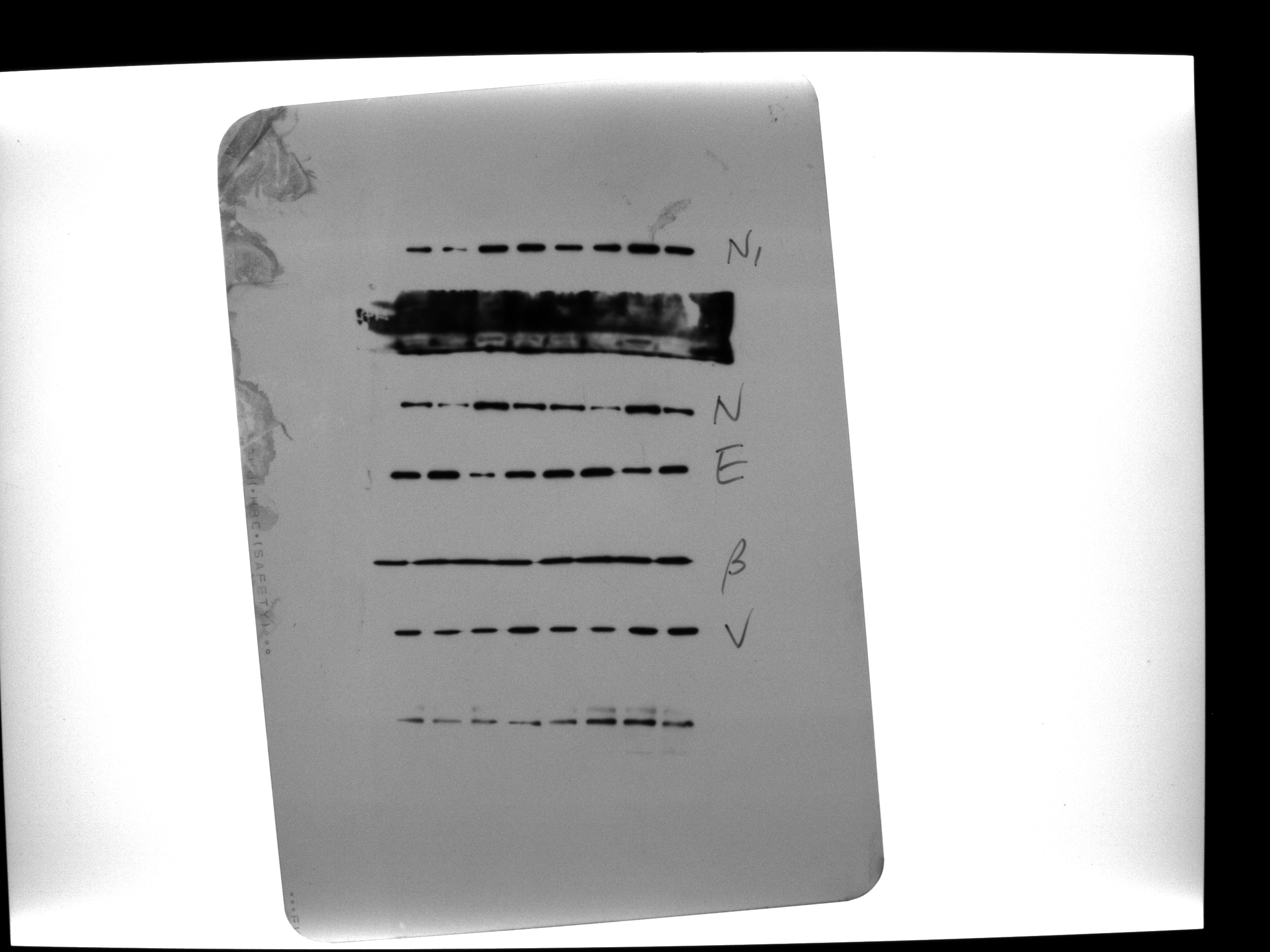

Supplement: Supplementary file 10 — Supplementary Information 10. [file 41598_2022_19326_MOESM10_ESM.jpg]

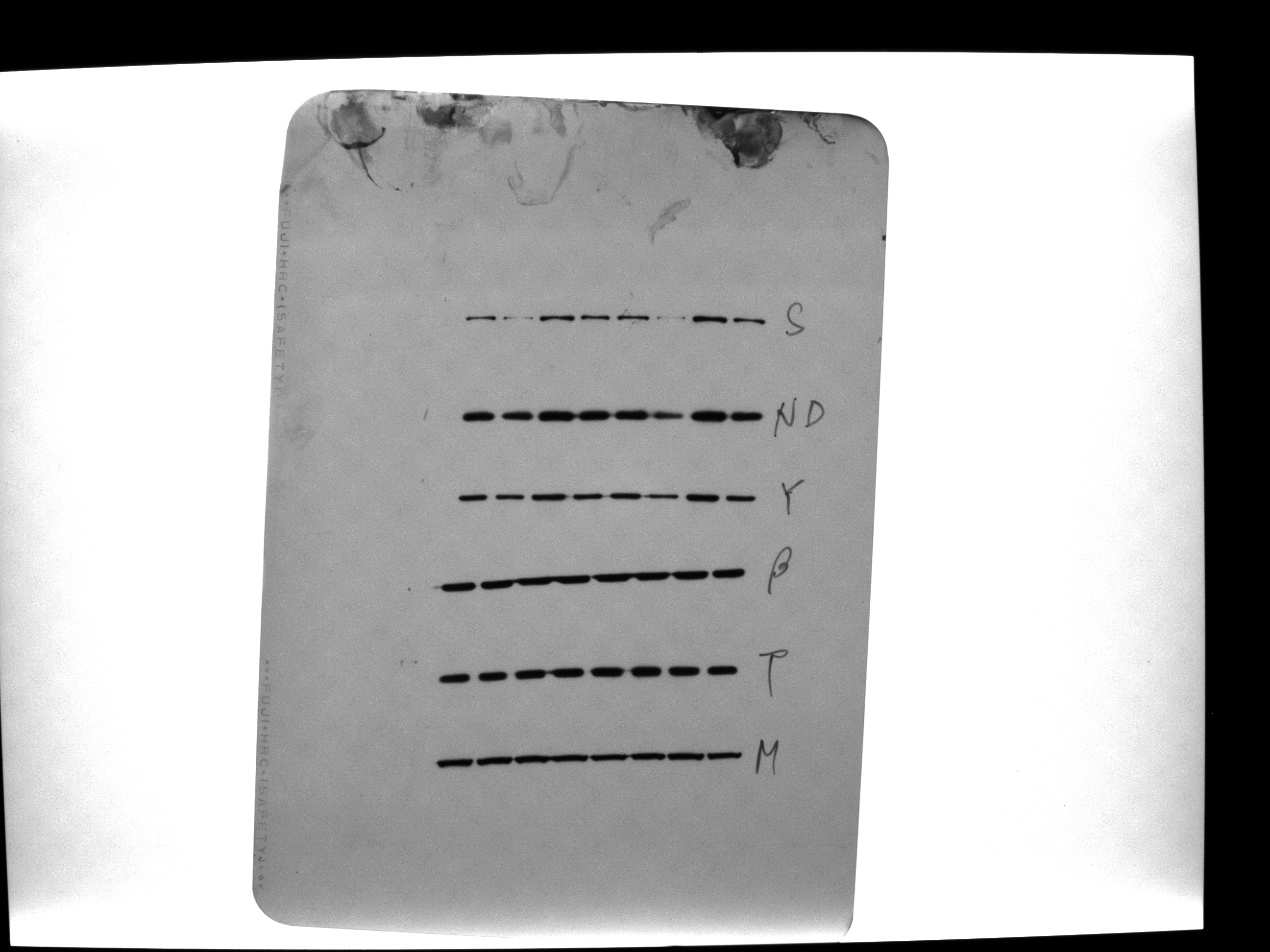

Supplement: Supplementary file 11 — Supplementary Information 11. [file 41598_2022_19326_MOESM11_ESM.jpg]

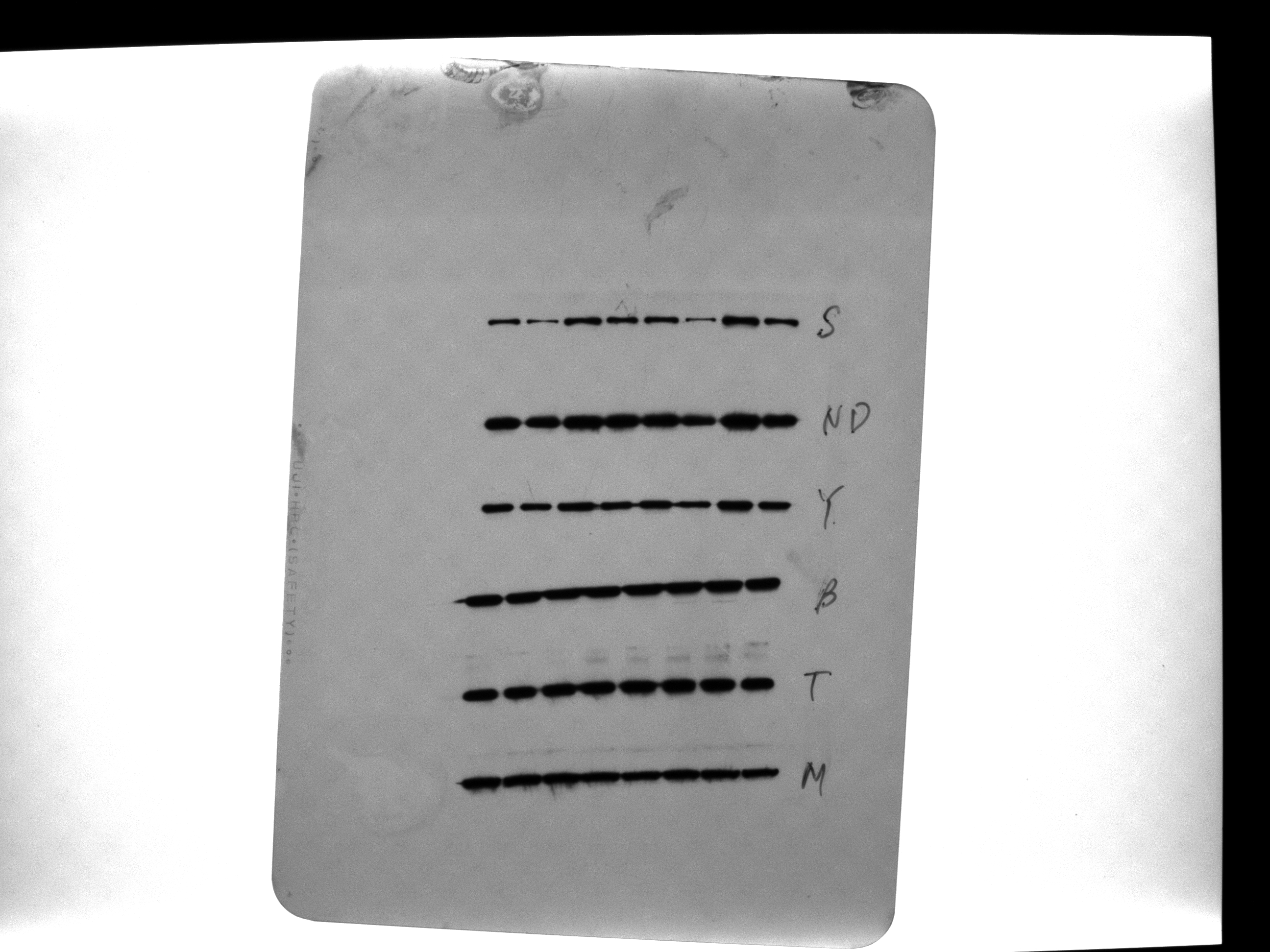

Supplement: Supplementary file 12 — Supplementary Information 12. [file 41598_2022_19326_MOESM12_ESM.jpg]

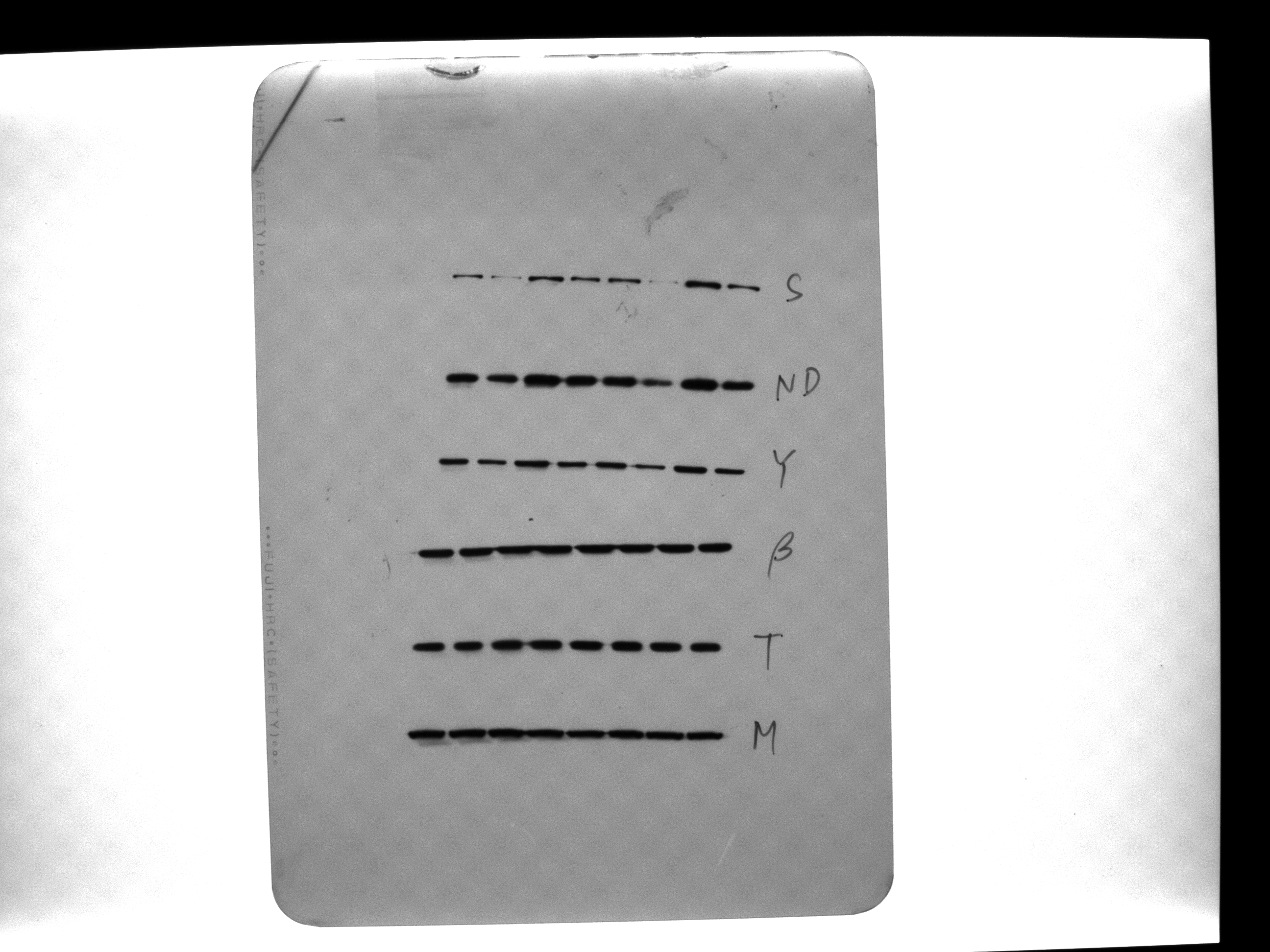

Supplement: Supplementary file 13 — Supplementary Information 13. [file 41598_2022_19326_MOESM13_ESM.jpg]
