# Supplementary material for: miR-30c plays diagnostic and prognostic roles and mediates epithelial–mesenchymal transition (EMT) and proliferation of gliomas by affecting Notch1
Source: Sci Rep. 2022 Sep 30;12:16404. doi: 10.1038/s41598-022-19326-x (PMC9525598; doi:10.1038/s41598-022-19326-x)
Supplement: Supplementary file 14 — Supplementary Information 14. [file 41598_2022_19326_MOESM14_ESM.doc]

**Figure S1 - uncropped Image of Figure 2C.** In the Western blot assay, the protein expression level of N-cadherin in the mimics group was lower than that in the NC group. Regarding the inhibitor group, the protein quantity of N-cadherin was higher than that of the inhibitor NC group.

**Figure S2 - uncropped Image of Figure 2C.** The protein expression level of Vimentin in the mimics group was lower than that in the NC group. The protein quantity of Vimentin in the inhibitor group was higher than that of the inhibitor NC group. β-actin was used as an internal reference.

**Figure S3 - uncropped Image of Figure 2C.** The protein expression level of E-cadherin in the mimics group was higher than that in the NC group. Regarding the inhibitor group, the protein expression level of E-cadherin was lower than that in the inhibitor NC group.

**Figure S4 - uncropped Image of Figure 4B.** After transfection of mimics into A172 and U251 cells, the expression levels of downstream target genes of Notch1, such as HES1 and HEY1, in the mimics group were significantly lower than those in the NC group. When inhibitor was transfected into glioma cells, the expression levels of the HES1 and HEY1 proteins were significantly higher than those in the inhibitor NC group. β-actin was used as the internal reference.

**Figure S5 - uncropped Image of Figure 4B.** When mimics was transfected into A172 and U251 cells, the expression level of NICD, which indicates the activation of Notch1 in the mimics group, was significantly lower than that in the NC group. After transfection of inhibitor into glioma cells, the expression level of the NICD protein was significantly higher than that in the inhibitor NC group.

**Figure S6 - uncropped Image of Figure 4B.** After transfection of mimics into glioma cells, the expression level of the Notch1 protein in the mimics group was significantly lower than that in the NC group. When inhibitor was transfected into glioma cells, the expression level of Notch1 protein in the inhibitor group was significantly higher than that in the inhibitor NC group.

**Figure S7 - uncropped Image of Figure 5B.** When shRNA-Notch1 were transfected into A172 and U251 cells, the protein expression level of E-cadherin was higher than that in the NC group.

**Figure S8 - uncropped Image of Figure 5B.** After transfection of shRNA-Notch1 into glioma cells, the protein level of N-cadherin in the shRNA-Notch1 group was lower than that in the NC group.

**Figure S9 - uncropped Image of Figure 5B.** The protein expression level of Vimentin in the shRNA-Notch1 group was lower than that in the NC group. β-actin was used as an internal reference.

**Figure S10 - uncropped Image of Figure 8A.** The miR-30c inhibitor raised the expression of Notch1 protein in A172 and U251 glioma cells, and shRNA-Notch1 attenuated this enhancement. The expression of the key factors N-cadherin increased, and the expression of E-cadherin decreased in the EMT process induced by the inhibitor, and shRNA-Notch1 attenuated these changes. But the expression of Vimentin was not affected in the cotransfection experiments.

**Figure S11 - uncropped Image of Figure 8A.** The miR-30c inhibitor enhanced the NICD protein in A172 and U251 glioma cells, and shRNA-Notch1 weakened this enhancement. β-actin was used as an internal reference.

**Figure S12 - uncropped Image of Figure 8A.** The miR-30c inhibitor improved the HES1 protein level in glioma cells, and shRNA-Notch1 attenuated this change.

**Figure S13 - uncropped Image of Figure 8A.** The miR-30c inhibitor enhanced the HEY1 protein level in glioma cells, and shRNA-Notch1 weakened this enhancement.
